# Supplementary material for: A Novel Ageladine A Derivative Acts as a STAT3 Inhibitor and Exhibits Potential Antitumor Effects
Source: Int J Mol Sci. 2023 May 16;24(10):8859. doi: 10.3390/ijms24108859 (PMC10219297; doi:10.3390/ijms24108859)
Supplement: Supplementary file 1 [file ijms-24-08859-s001.zip › ijms-2296089-supplementary.pdf]

Supporting Information

Compounds **2**–**10** were prepared according to the procedure published by Zhong *et al,* Naoki *et al and* Song *et al,* the spectroscopic data for the intermediates were identical to those described in the literature [34].

1. General Procedure for Compounds **9**–**12**.

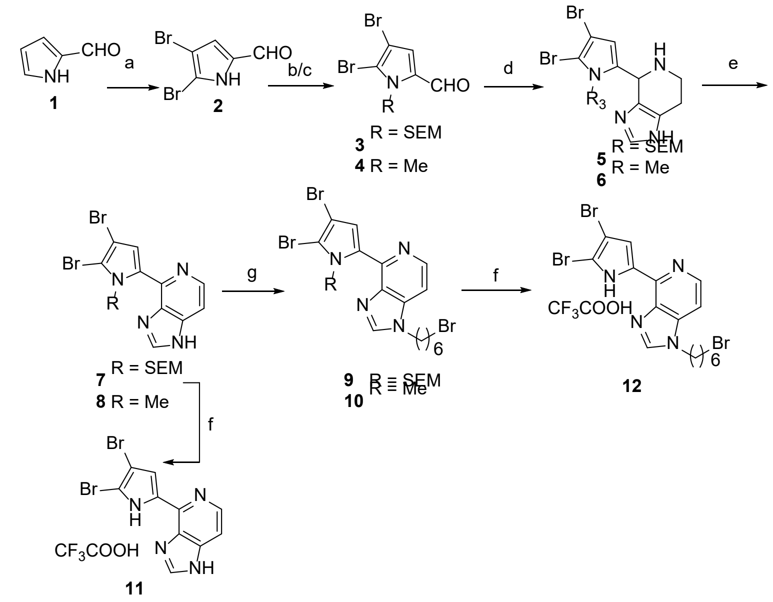

Scheme S1. Synthesis of Compounds **9–12**. Reagents and conditions: (a) NBS, THF, 0 °C ~ rt, 3 h; (b) SEMCl, *t*-BuOK, rt, overnight; (c) CH_3I, K_2CO_3, DMF, rt, overnight; (d) Histamine dihydrochloride, EtOH, 80 °C, 12 h; (e) IBX, DMSO, rt, 2 h; MnO_2, DCM, rt, 2.5 h; (f) Boron (tri) fluoride etherate, DCM, 15 h, trifluoroacetic acid; (g) Cs_2CO_3, DMF, $Br(CH_2)_6Br$, rt, 3 h.

To a solution of **5–6** (1 mmol) in DMSO was added IBX (1.5 mmol), and the mixture was stirred at room temperature for 2 h. The reaction was quenched by adding H_2O and an aqueous NaOH solution (1.0 mol/L, 300 mL), and the mixture was extracted with EtOAc. The organic extracts were combined, washed with H_2O and brine successively, dried over anhydrous $MgSO_4$, filtered, and then concentrated in vacuo. To a solution of the residue in CH_2Cl_2 was added activated MnO_2 (5.0 mol), and the mixture was stirred at room temperature for 2.5 h. The reaction mixture was filtered, and the filtrate was concentrated in vacuo. The residue was purified by column chromatography (petroleum ether/ethyl acetate 4:1) to give **7**–**8**.

To a stirred solution of **7–8** (0.75 mmol) in DMF (30 mL) was added Cs_2CO_3 (2.25 mmol) and appropriate dibromoalkyl (2.25 mmol) at room temperature for 3 h. The reaction mixture was extracted with EtOAc. The combined extracts were washed with H_2O and brine, dried over anhydrous $MgSO_4$, filtered, and then concentrated in vacuo. The reaction residue was subjected to preliminary purification on a silica gel column (petroleum ether/ethyl acetat 8:1) to obtain **9**–**10**.

To a solution of **7** (0.20 mmol) in CH_2Cl_2 (5.0 mL) was added BF_3-OEt_2 (2.1 mmol) under a nitrogen atmosphere, and the mixture was stirred at room temperature for 15 h. The reaction was quenched by adding saturated sodium bicarbonate solution, and the reaction mixture was extracted with EtOAc. The combined extracts were washed with H_2O and brine, dried over anhydrous $MgSO_4$, filtered, and then concentrated in vacuo. The residue was dissolved in MeOH and TFA (0.10 mL) was added to the MeOH solution. The acidic methanolic solution was concentrated in vacuo.

Trituration of the residue with CH_2Cl_2 gave **11**.

Treatments of **9** (100 mg, 0.16 mmol) with BF_3-OEt_2 (0.2 mL, 1.6 mmol) and TFA (0.10 mL) carried out in the same manner as described for **7** gave **12.**

4-[4,5-dibromo-1-[[2-(trimethylsilyl)ethoxy]methyl]-1H-pyrrol-2-yl]-3H-imidazo[4,5-c]pyridine (**7**)

Yellow powder (235 mg, 50%); ^{1}H NMR (500 MHz, DMSO-d_6) δ 8.45 (s, 1H), 8.32 (d, J = 5.5 Hz, 1H), 7.57–7.46 (m, 2H), 6.13 (s, 2H), 3.24 (t, J = 7.9 Hz, 2H), 0.59 (t, J = 8.0 Hz, 2H), -0.30 (s, 9H); ^{13}C NMR (125 MHz, DMSO-d_6) δ 144.50, 140.39, 131.82, 117.24, 109.99, 109.35, 100.03, 75.29, 65.17, 17.45, -1.25. HRMS (ESI+) *m/z*: Calcd for $C_{16}H_{21}Br_2N_4OSi$; $[M+H]^+$ 470.9846, Found 470.9845.

4-(4,5-dibromo-1-methyl-1H-pyrrol-2-yl)-1H-imidazo[4,5-c]pyridine (**8**)

Yellow powder (140 mg, yield 40%); ^{1}H NMR (400 MHz, Methanol-d_4) δ 8.40 (d, J = 5.7 Hz, 1H), 8.38 (s, 1H), 7.62 (d, J = 5.7 Hz, 1H), 6.94 (s, 1H), 3.87 (s, 3H); ^{13}C NMR (101 MHz, Methanol-d_4) δ 144.38, 144.30, 140.25, 130.42, 129.46, 114.84, 114.18, 108.37, 98.10, 34.88; HRMS (ESI$^+$) *m/z*: Calcd for $C_{11}H_9Br_2N_4$; $[M+H]^+$ 354.9188, Found 354.9205.

1-(6-bromohexyl)-4-(4,5-dibromo-1-((2-(trimethylsilyl)ethoxy)methyl)-1H-pyrrol-2-yl)-1H-imidazo[4,5-c]pyridine (**9**)

Yellow adhesive liquid (282 mg, yield 60%); ^{1}H NMR (500 MHz, Chloroform-d) δ 8.42 (d, J = 5.6 Hz, 1H), 7.95 (s, 1H), 7.52 (s, 1H), 7.24 (d, J = 5.6 Hz, 1H), 6.19 (s, 2H), 4.20 (t, J = 7.1 Hz, 2H), 3.41 – 3.34 (m, 4H), 1.92 (dt, J = 15.0, 7.3 Hz, 2H), 1.86 (dd, J = 14.3, 7.3 Hz, 2H), 1.51 (dd, J = 15.4, 7.7 Hz, 2H), 1.38 (dd, J = 15.4, 8.0 Hz, 2H), 0.75–0.70 (m, 2H), -0.23 (s, 9H); ^{13}C NMR (125 MHz, Chloroform-d) δ 143.53, 140.81, 139.37, 137.73, 131.01, 118.22, 109.61, 103.67, 100.82, 75.40, 65.43, 45.12, 33.41, 32.30, 29.74, 27.49, 25.90, 17.62, -1.65; HRMS (ESI$^+$) *m/z*: Calcd for $C_{22}H_{32}Br_3N_4OSi$; $[M+H]^+$ 638.9841, Found 638.9829.

1-(6-bromohexyl)-4-(4,5-dibromo-1-methyl-1H-pyrrol-2-yl)-1H-imidazo[4,5-c]pyridine (**10**)

Yellow adhesive liquid (240 mg, yield 62%); ^{1}H NMR (500 MHz, Chloroform-d) δ 8.42 (d, J = 5.6 Hz, 1H), 7.94 (s, 1H), 7.41 (s, 1H), 7.22 (d, J = 5.6 Hz, 1H), 4.20 (t, J = 7.1 Hz, 2H), 4.07 (s, 3H), 3.39 (t, J = 6.6 Hz, 2H), 1.92 (dt, J = 14.9, 7.3 Hz, 2H), 1.87–1.80 (m, 2H), 1.51 (dt, J = 15.2, 7.4 Hz, 2H), 1.36 (dt, J = 15.5, 7.7 Hz, 2H); ^{13}C NMR (125 MHz, Chloroform-d) δ 143.75, 143.46, 141.02, 139.23, 137.79, 130.83, 116.80, 109.48, 103.48, 98.77, 45.12, 36.31, 33.49, 32.31, 29.73, 27.50, 25.89; HRMS (ESI$^+$) *m/z*: Calcd for $C_{17}H_{20}Br_3N_4$; $[M+H]^+$ 520.0880, Found 520.0890.

4-(4,5-dibromo-1H-pyrrol-2-yl)-1H-imidazo[4,5-c]pyridine -TFA (**11**)

Yellow powder (77 mg, yield 85%); ^{1}H NMR (500 MHz, DMSO-d_6) δ 13.29 (s, 1H),8.73 (s, 1H), 8.32 (d, J = 6.2 Hz, 1H), 7.82 (s, 1H), 7.73 (d, J = 6.2 Hz, 1H); ^{13}C NMR (125 MHz, DMSO-d_6) δ 158.79, 158.53, 147.50, 142.36, 134.66, 118.91, 107.82, 100.88; HRMS (ESI$^+$) *m/z*: Calcd for $C_{10}H_6Br_2N_4$; $[M+H]^+$ 340.9032, Found 340.9042.

1-(6-bromohexyl)-4-(4,5-dibromo-1H-pyrrol-2-yl)-1H-imidazo[4,5-c]pyridine -TFA (**12**)

Yellow amorphous solid (89 mg, 90%); ^{1}H NMR (500 MHz, Methanol-d_4) δ 8.66 (s, 1H), 8.30 (d, J = 6.5 Hz, 1H), 7.86 (d, J = 6.5 Hz, 1H), 7.43 (s, 1H), 4.43 (t, J = 7.3 Hz, 2H), 3.43 (t, J = 6.7 Hz, 2H), 1.97 (dt, J = 15.0, 7.5 Hz, 2H), 1.88–1.81 (m, 2H), 1.52 (dt, J = 14.9, 7.3 Hz, 2H), 1.41 (dt, J = 15.3, 7.5 Hz, 2H); ^{13}C NMR (126 MHz, Methanol-d_4) δ 148.79, 142.63, 135.89, 135.09, 133.86, 124.44, 116.82, 109.05, 105.43, 101.59, 45.47, 32.74, 32.24, 29.41, 27.19, 25.38; HRMS (ESI$^+$) *m/z*: Calcd for $C_{16}H_{18}Br_3N_4$; $[M+H]^+$ 508.9015, Found 508.9024.

2. General Procedure for Compounds **13**–**25**.

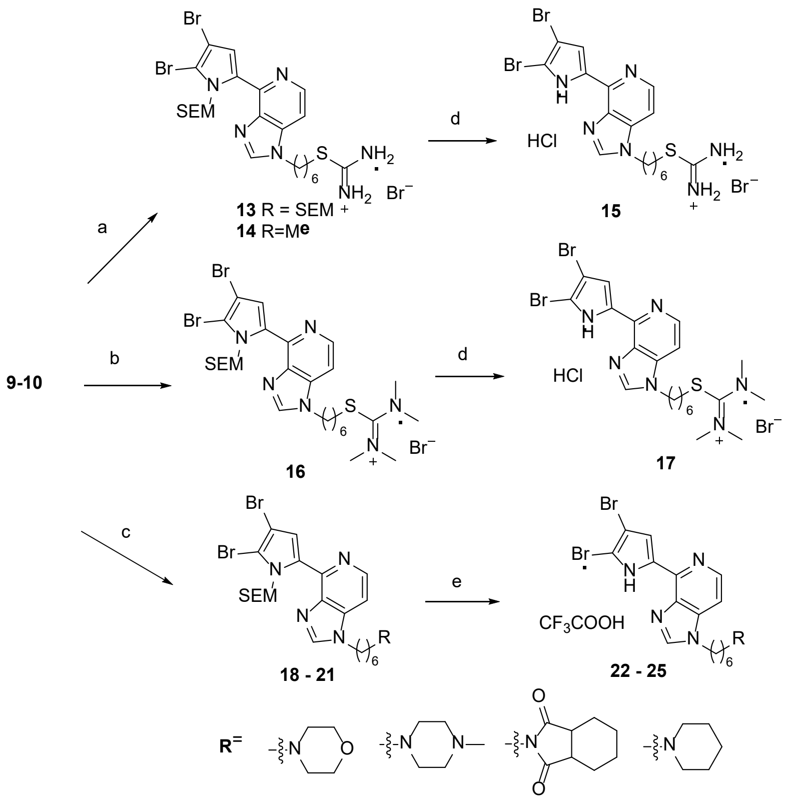

Scheme S2. Synthesis of Compounds **13–25**. Reagents and conditions:(a) Thiourea, THF, 70 °C, overnight; (b) 1,1,3,3-Tetramethylthiourea, THF, 70 °C, overnight; (c) Heterocycle, K_2CO_3, DMF, rt, overnight; (d) HCl, MeOH, 70 °C, overnight; (e)Boron (tri) fluoride etherate, DCM, 15 h, trifluoroacetic acid.

Compounds **9–10** (0.16 mmol) were mixed with thiourea (1.6 mmol) or 1,1,3,3-tetramethylthiourea (1.6 mmol), respectively, in THF (20 mL) and refluxed overnight. The organic solvent was distilled off in vacuo and the reaction residue was subjected to silica gel column chromatography to obtain compounds **13–14** and **16**.

HCl (1 mL) was added to a solution of **13–14** and **16** (0.05 mmol) in MeOH (20 mL) respectively, and the reaction was stirred at room temperature overnight. The solvent was evaporated under vacuum to get compounds **15** and **17** as brown amorphous solid.

To a stirred solution of **10** (0.16 mmol) in DMF (30 mL) was added K_2CO_3 (0.32 mmol) and appropriate heterocycle (0.32 mmol) at room temperature for 3 h. The reaction mixture was extracted with EtOAc. The combined extracts were washed with H_2O and brine, dried over anhydrous $MgSO_4$, filtered, and then concentrated in vacuo. The reaction residue was subjected to preliminary purification on a silica gel column (petroleum ether/ethyl acetate 25:1) to obtain **18–21**.

Similar treatments of **18–21** (0.12 mmol) with BF_3-OEt_2 (1.2 mmol) and TFA (0.10 mL) to those described for **11** gave **22–25** as a pale-yellow powder after trituration with CH_2Cl_2-MeOH.

2-(6-(4-(4,5-dibromo-1-((2-(trimethylsilyl)ethoxy)methyl)-1H-pyrrol-2-yl)-1H-imidazo[4,5-c]pyridin-1-yl)hexyl)isothiouronium hydrobromide (**13**)

Yellow powder (45 mg, yield 45%); ^1H NMR (500 MHz, DMSO-d_6) δ 9.08 (s, 4H), 8.47 (s, 1H), 8.35 (d, J = 5.5 Hz, 1H), 7.65 (d, J = 5.5 Hz, 1H), 7.54 (s, 1H), 6.17 (s, 2H), 4.30 (t, J = 6.9 Hz, 2H), 3.28–3.23 (m, 2H), 3.12 (t, J = 7.3 Hz, 2H), 1.79 (dt, J = 14.3, 7.0 Hz, 2H), 1.56 (dt, J = 14.7, 7.4 Hz, 2H), 1.42–1.35 (m, 2H), 1.29–1.23 (m, 2H), 0.63–0.58 (m, 2H), -0.30 (s, 9H); ^{13}C NMR (125

MHz, DMSO-d_6) δ 170.35, 145.91, 142.22, 140.54, 139.79, 137.56, 131.89, 117.61, 109.46, 105.62, 99.99, 75.31, 65.11, 44.71, 30.39, 29.66, 28.70, 27.70, 25.85, 17.46, -1.25; HRMS (ESI$^+$) *m/z*: Calcd for $C_{23}H_{35}Br_2N_6OSSi$; $[M+H]^+$ 633.0683, Found 633.0692.

1-(6-(4-(4,5-dibromo-1-methyl-1H-pyrrol-2-yl)-1H-imidazo[4,5-c]pyridin-1-yl)hexyl)isothiouronium hydrobromide (**14**)

Yellow powder (33 mg, yield 40%); ^{1}H NMR (500 MHz, DMSO-d_6) δ 9.06 (s, 4H), 8.44 (s, 1H), 8.34 (d, *J* = 5.5 Hz, 1H), 7.60 (d, *J* = 5.5 Hz, 1H), 7.52 (s, 1H), 4.28 (t, *J* = 6.8 Hz, 2H), 4.07 (s, 3H), 3.11 (t, *J* = 7.2 Hz, 2H), 1.82–1.75 (m, 2H), 1.55 (dt, *J* = 14.4, 7.3 Hz, 2H), 1.37 (dt, *J* = 14.7, 7.4 Hz, 2H), 1.27–1.22 (m, 2H); ^{13}C NMR (125 MHz, DMSO-d_6) δ 170.33, 145.75, 142.39, 140.64, 139.66, 137.37, 131.42, 116.62, 109.34, 105.34, 97.96, 44.71, 36.92, 30.38, 29.63, 28.68, 27.69, 25.86; HRMS (ESI$^+$) *m/z*: Calcd for $C_{18}H_{24}Br_2N_6S$; $[M+H]^+$ 515.0046, Found 515.0054..

1-(6-(4-(4,5-dibromo-1H-pyrrol-2-yl)-1H-imidazo[4,5-c]pyridin-1-yl)hexyl)isothiouronium hydrochloride hydrobromide (**15**)

Yellow powder (28 mg, yield 91%); ^{1}H NMR (400 MHz, Methanol-d_4) δ 8.81 (s, 1H), 8.35 (d, *J* = 6.7 Hz, 1H), 8.04 (d, *J* = 6.7 Hz, 1H), 7.60 (s, 1H), 4.52 (t, *J* = 7.2 Hz, 2H), 3.18 (t, *J* = 7.3 Hz, 2H), 2.06 – 1.96 (m, 2H), 1.80–1.70 (m, 2H), 1.61–1.52 (m, 2H), 1.47 (dt, *J* = 14.5, 7.5 Hz, 2H); ^{13}C NMR (100 MHz, Methanol-d_4) δ 171.62, 149.72, 144.46, 143.36, 135.01, 134.88, 132.37, 123.04, 117.81, 117.49, 110.38, 105.92, 105.87, 102.07, 30.31, 29.43, 28.12, 27.55, 25.69; HRMS (ESI$^+$) *m/z*: Calcd for $C_{17}H_{21}Br_2N_6S$; $[M+H]^+$ 500.9889, Found 500.9901.

1-(6-(4-(4,5-dibromo-1-((2-(trimethylsilyl)ethoxy)methyl)-1H-pyrrol-2-yl)-1H-imidazo[4,5-c]pyridin-1-yl)hexyl)-1,1,3,3-tetramethylisothiouronium hydrobromide (**16**)

Yellow powder (52 mg, yield 48%); ^{1}H NMR (500 MHz, DMSO-d_6) δ 8.48 (s, 1H), 8.35 (d, *J* = 5.5 Hz, 1H), 7.65 (d, *J* = 5.5 Hz, 1H), 7.55 (s, 1H), 6.18 (s, 2H), 4.31 (t, *J* = 6.9 Hz, 2H), 3.30–3.25 (m, 2H), 3.21 (s, 12H), 3.00 (t, *J* = 7.3 Hz, 2H), 1.80 (dd, *J* = 14.7, 7.2 Hz, 2H), 1.55 (dt, *J* = 14.8, 7.4 Hz, 2H), 1.38 (dt, *J* = 14.8, 7.6 Hz, 2H), 1.25 (dt, *J* = 15.2, 7.7 Hz, 2H), 0.65–0.59 (m, 2H), -0.29 (s, 9H); ^{13}C NMR (125 MHz, DMSO-d_6) δ 174.56, 145.94, 142.21, 140.53, 139.79, 137.54, 131.86, 117.63, 109.51, 105.66, 99.99, 75.34, 65.12, 44.72, 43.91, 34.16, 29.64, 29.51, 27.83, 25.91, 17.47, -1.23; HRMS (ESI$^+$) *m/z*: Calcd for $C_{27}H_{43}Br_2N_6OSSi$; $[M+H]^+$ 689.1309, Found 689.1308.

1-(6-(4-(4,5-dibromo-1H-pyrrol-2-yl)-1H-imidazo[4,5-c]pyridin-1-yl)hexyl)-1,1,3,3-tetramethylisothiouronium hydrochloride hydrobromide (**17**)

Yellow powder (27 mg, yield 80%); ^{1}H NMR (400 MHz, Methanol-d_4) δ 8.82 (s, 1H), 8.36 (s, 1H), 8.06 (s, 1H), 7.59 (s, 1H), 4.53 (s, 2H), 3.37 (s, 3H), 3.10 (s, 2H), 2.03 (s, 2H), 1.75 (s, 2H), 1.53 (d, *J* = 28.7 Hz, 4H); ^{13}C NMR (100 MHz, Methanol-d_4) δ 176.00, 149.90, 143.44, 135.10, 134.97, 132.53, 123.08, 117.78, 110.45, 106.15, 102.11, 45.83, 43.15, 34.30, 29.55, 29.31, 27.87, 25.89; HRMS (ESI$^+$) *m/z*: Calcd for $C_{21}H_{29}Br_2N_6S$; $[M+H]^+$ 555.0536, Found 555.0527.

1-(6-(4-(4,5-dibromo-1-((2-(trimethylsilyl)ethoxy)methyl)-1H-pyrrol-2-yl)-1H-imidazo[4,5-c]pyridin-1-yl)hexyl)morpholine (**18**)

Yellow adhesive liquid (81 mg, yield 80%); ^{1}H NMR (500 MHz, Chloroform-*d*) δ 8.40 (d, *J* = 5.5 Hz, 1H), 7.94 (s, 1H), 7.51 (s, 1H), 7.22 (d, *J* = 5.5 Hz, 1H), 6.18 (s, 3H), 4.18 (t, *J* = 7.1 Hz, 3H), 3.70 (t, *J* = 4.5 Hz, 4H), 3.49–3.28 (m, 2H), 2.41 (s, 4H), 2.32 – 2.27 (m, 2H), 1.93–1.86 (m, 2H), 1.52 – 1.44 (m, 2H), 1.36 (s, 4H), 0.75–0.69 (m, 2H), -0.23 (s, 9H); ^{13}C NMR (125 MHz, Chloroform-*d*) δ 143.62, 143.58, 140.98, 139.43, 137.85, 131.31, 118.21, 103.77, 75.46, 67.01, 65.51, 58.89, 53.83, 45.31, 29.94, 26.99, 26.77, 26.41, 17.72, -1.56; HRMS (ESI$^+$) *m/z*: Calcd for $C_{26}H_{40}Br_2N_5O_2Si$; $[M+H]^+$ 640.1313, Found 640.1315.

1-(6-(4,5-dibromo-1-((2-(trimethylsilyl)ethoxy)methyl)-1H-pyrrol-2-yl)-1-(6-(4-methylpiperazin-1-yl)hexyl)-1H-imidazo[4,5-c]pyridine (**19**)

Yellow adhesive liquid (84 mg, yield 80%); ^{1}H NMR (500 MHz, Chloroform-*d*) δ 8.40 (d, J = 5.5 Hz, 1H), 7.94 (s, 1H), 7.51 (s, 1H), 7.22 (d, J = 5.6 Hz, 1H), 6.18 (s, 2H), 4.18 (t, J = 7.1 Hz, 2H), 3.40 – 3.31 (m, 2H), 2.47 (s, 4H), 2.34–2.30 (m, 2H), 2.29 (s, 3H), 1.92 (d, J = 29.2 Hz, 6H), 1.48 (s, 2H), 1.36 (s, 4H), 0.75–0.69 (m, 2H), -0.23 (s, 9H); ^{13}C NMR (126 MHz, Chloroform-*d*) δ 143.50, 140.88, 139.33, 137.74, 135.59, 131.21, 118.06, 109.44, 103.71, 100.77, 75.34, 65.41, 58.32, 54.92, 53.04, 45.90, 45.22, 29.83, 26.95, 26.66, 26.58, 17.61, -1.65; HRMS (ESI$^+$) *m/z*: Calcd for $C_{27}H_{43}Br_2N_6OSi$; [M+H]$^+$ 653.1629, Found 653.1634.

1-(6-(4-(4,5-dibromo-1-((2-(trimethylsilyl)ethoxy)methyl)-1H-pyrrol-2-yl)-1H-imidazo[4,5-c]pyridin-1-yl)hexyl)isoindoline-1,3-dione (**20**)

White solid (95 mg, yield 85%); ^{1}H NMR (500 MHz, Chloroform-*d*) δ 8.39 (d, J = 5.6 Hz, 1H), 7.95 (s, 1H), 7.84 (q, J = 3.0 Hz, 2H), 7.71 (dd, J = 5.4, 3.1 Hz, 2H), 7.50 (s, 1H), 7.22 (d, J = 5.6 Hz, 1H), 6.17 (s, 2H), 4.17 (t, J = 7.2 Hz, 2H), 3.68 (t, J = 7.1 Hz, 2H), 3.36 (t, J = 8.3 Hz, 2H), 1.93–1.86 (m, 2H), 1.72–1.65 (m, 2H), 1.41 (dt, J = 7.1, 3.4 Hz, 4H), 0.71 (t, J = 8.3 Hz, 2H), -0.23 (s, 9H); ^{13}C NMR (125 MHz, Chloroform-*d*) δ 168.41, 143.48, 140.89, 139.32, 137.76, 133.96, 132.04, 131.23, 123.21, 118.06, 109.41, 103.70, 100.78, 65.40, 45.19, 37.58, 29.75, 29.68, 28.29, 26.23, 26.20, 17.62, -1.66; HRMS (ESI$^+$) *m/z*: Calcd for $C_{30}H_{36}Br_2N_5O_3Si$; [M+H]$^+$ 700.0949, Found 700.0943.

1-(6-(4,5-dibromo-1-((2-(trimethylsilyl)ethoxy)methyl)-1H-pyrrol-2-yl)-1-(6-(piperidin-1-yl)hexyl)-1H-imidazo[4,5-c]pyridine (**21**)

Yellow adhesive liquid (81 mg, yield 79%); ^{1}H NMR (500 MHz, Methanol-*d*$_4$) δ 8.42 (d, J = 5.6 Hz, 1H), 8.35 (s, 1H), 7.63 (d, J = 5.6 Hz, 1H), 7.14 (s, 1H), 5.98 (s, 2H), 4.37 (t, J = 7.0 Hz, 2H), 3.23 (t, J = 8.0 Hz, 2H), 3.12 (s, 2H), 3.00–2.94 (m, 2H), 1.98–1.92 (m, 2H), 1.82 (s, 4H), 1.74–1.62 (m, 4H), 1.42 (s, 4H), 0.61 (t, J = 8.0 Hz, 2H), -0.25 (s, 9H); ^{13}C NMR (125 MHz, Methanol-*d*$_4$) δ 145.53, 142.59, 140.68, 139.84, 137.49, 131.34, 116.73, 108.45, 105.07, 100.04, 75.16, 65.20, 56.87, 52.96, 44.72, 29.29, 25.84, 25.82, 23.68, 23.07, 21.53, 16.95, -2.97; HRMS (ESI$^+$) *m/z*: Calcd for $C_{27}H_{42}Br_2N_5OSi$; [M+H]$^+$ 638.1520, Found 638.1519.

1-(6-(4-(4,5-dibromo-1H-pyrrol-2-yl)-1H-imidazo[4,5-c]pyridin-1-yl)hexyl)morpholine-TFA (**22**)

Yellow powder (66 mg, yield 85%); ^{1}H NMR (500 MHz, Methanol-*d*$_4$) δ 8.67 (s, 1H), 8.32 (d, J = 6.5 Hz, 1H), 7.88 (d, J = 6.5 Hz, 1H), 7.49 (s, 1H), 4.45 (t, J = 7.2 Hz, 2H), 4.04 (d, J = 12.0 Hz, 2H), 3.75 (t, J = 11.5 Hz, 2H), 3.47 (d, J = 12.3 Hz, 2H), 3.15–3.06 (m, 4H), 2.02–1.94 (m, 2H), 1.75 (dt, J = 15.6, 7.7 Hz, 2H), 1.48–1.42 (m, 4H); ^{13}C NMR (125 MHz, Methanol-*d*$_4$) δ 149.43, 143.93, 143.17, 135.35, 135.12, 132.92, 123.49, 117.42, 105.75, 101.98, 56.66, 52.87, 45.50, 29.32, 25.70, 25.67, 23.44, 22.87, 21.31; HRMS (ESI$^+$) m/z: Calcd for $C_{20}H_{26}Br_2N_5O$; [M+H]$^+$ 510.0499, Found 510.0489.

1-(6-(4,5-dibromo-1H-pyrrol-2-yl)-1-(6-(4-methylpiperazin-1-yl)hexyl)-1H-imidazo[4,5-c]pyridine -TFA (**23**)

Yellow powder (65 mg, yield 85%); ^{1}H NMR (500 MHz, Methanol-*d*$_4$) δ 8.81 (s, 1H), 8.34 (d, J = 6.6 Hz, 1H), 8.04 (d, J = 6.6 Hz, 1H), 7.63 (s, 1H), 4.50 (t, J = 7.1 Hz, 2H), 3.72 (s, 8H), 3.30 – 3.25 (m, 3H), 3.01 (s, 3H), 2.04–1.98 (m, 2H), 1.87–1.79 (m, 2H), 1.54 – 1.45 (m, 4H); ^{13}C NMR (500 MHz, Methanol-*d*$_4$) δ 160.82, 160.53, 149.67, 143.29, 134.99, 132.42, 123.11, 117.97, 115.11, 110.21, 105.96, 101.98, 56.22, 49.84, 45.55, 41.95, 29.19, 25.47, 25.41, 23.19; HRMS (ESI$^+$) *m/z*:

Calcd for $C_{21}H_{29}Br_2N_6$; $[M+H]^+$ 523.0815, Found 525.0805.

1-(6-(4-(4,5-dibromo-1H-pyrrol-2-yl)-1H-imidazo[4,5-c]pyridin-1-yl)hexyl)isoindoline-1,3-dione -TFA (**24**)

Yellow powder (63 mg, yield 83%); ^{1}H NMR (500 MHz, Methanol-*d*$_4$) δ 8.69 (s, 1H), 8.31 (d, *J* = 6.4 Hz, 1H), 7.90 (d, *J* = 6.3 Hz, 1H), 7.82–7.75 (m, 4H), 7.46 (s, 1H), 4.44 (t, *J* = 7.1 Hz, 2H), 3.63 (t, *J* = 7.0 Hz, 2H), 2.00–1.92 (m, 2H), 1.70–1.63 (m, 2H), 1.47–1.36 (m, 4H); ^{13}C NMR (125 MHz, Methanol-*d*$_4$) δ 168.41, 149.12, 142.91, 135.13, 133.92, 133.33, 131.86, 123.98, 122.60, 116.97, 105.57, 101.79, 45.55, 37.05, 29.31, 27.76, 25.81, 25.63; HRMS (ESI$^+$) *m/z*: Calcd for $C_{24}H_{22}Br_2N_5O_2$; $[M+H]^+$ 570.0135, Found 570.0125.

3. General Procedure for Compounds **26–28.**

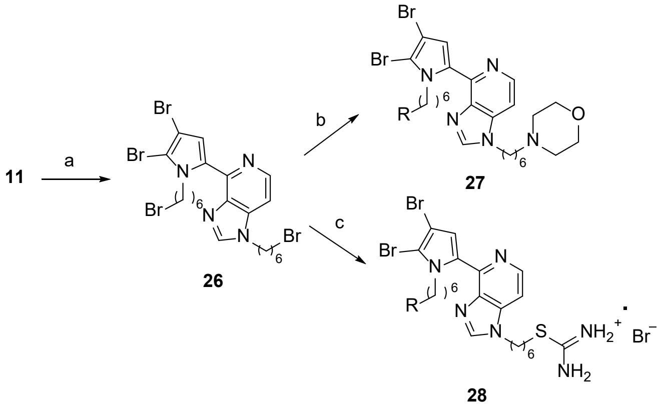

Scheme S3. Synthesis of compounds **26–28**. Reagents and conditions: (a) Cs_2CO_3, DMF, $Br(CH_2)_6Br$, rt, overnight; (b) Morpholine, K_2CO_3, DMF, rt, overnight; (c) Thiourea, THF, 70 °C, overnight.

To a stirred solution of **11** (500 mg, 1.1 mmol) in DMF (30 mL) was added Cs_2CO_3 (1.43 g, 4.38 mmol) and 1,6-dibromohexane (0.67 mL, 4.38 mmol) at room temperature for 3 h. The reaction mixture was extracted with EtOAc. The combined extracts were washed with H_2O and brine, dried over anhydrous $MgSO_4$, filtered, and then concentrated in vacuo. The reaction residue was subjected to preliminary purification on a silica gel column (petroleum ether/ethyl acetate 10:1) to obtain **26**.

The same treatments of **26** (100 mg, 0.15 mmol) with K_2CO_3 ((84 mg, 0.6 mmol) and morpholine (53 mg, 0.6 mmol) as those described for **18** gave **27**.

Treatments of **26** (100 mg, 0.15 mmol) with thiourea (103 mg, 1.35 mmol) carried out in the same manner as described for **16** gave **28**.

1-(6-(4,5-dibromo-1H-pyrrol-2-yl)-1-(6-(piperidin-1-yl)hexyl)-1H-imidazo[4,5-c]pyridine - TFA (**25**)

Yellow powder (68 mg, yield 85%); ^{1}H NMR (400 MHz, Methanol-*d*$_4$) δ 8.76 (s, 1H), 8.35 (d, *J* = 6.5 Hz, 1H), 7.98 (d, *J* = 6.5 Hz, 1H), 7.54 (s, 1H), 4.49 (t, *J* = 7.2 Hz, 2H), 3.53 (d, *J* = 12.4 Hz, 2H), 3.12–3.04 (m, 2H), 2.91 (t, *J* = 12.4 Hz, 2H), 2.10–1.68 (m, 12H); ^{13}C NMR (100 MHz, Methanol-*d*$_4$) δ 149.43, 143.93, 143.17, 135.35, 135.12, 132.92, 123.49, 117.42, 110.06, 109.05, 105.75, 101.98, 56.66, 52.87, 45.50, 29.32, 25.70, 25.67, 23.44, 22.87, 21.31; HRMS (ESI$^+$) *m/z*: Calcd for $C_{21}H_{28}Br_2N_5$; $[M+H]^+$ 508.0706, Found 508.0698.

1-(6-bromohexyl)-4-(4,5-dibromo-1-(6-bromohexyl)-1H-pyrrol-2-yl)-1H-imidazo[4,5-c]pyridine (**26**)

Yellow powder (367 mg, yield 50%). ^{1}H NMR (600 MHz, Chloroform-*d*) δ 8.39 (d, *J* = 5.6 Hz, 1H),

7.94 (s, 1H), 7.51 (s, 1H), 7.21 (d, *J* = 5.6 Hz, 1H), 4.72–4.64 (m, 2H), 4.19 (t, *J* = 7.1 Hz, 2H), 3.38 (t, *J* = 6.6 Hz, 2H), 3.30 (t, *J* = 6.9 Hz, 2H), 1.92 (dt, *J* = 14.9, 7.3 Hz, 2H), 1.86–1.81 (m, 2H), 1.74 (dd, *J* = 14.5, 7.3 Hz, 2H), 1.69 (dd, *J* = 14.1, 6.6 Hz, 2H), 1.66–1.60 (m, 2H), 1.50 (dt, *J* = 15.3, 7.5 Hz, 2H), 1.39–1.33 (m, 4H); ^{13}C NMR (150 MHz, Chloroform-*d*) δ 143.78, 143.49, 141.00, 139.37, 137.66, 130.21, 117.49, 108.82, 103.54, 99.24, 48.13, 45.23, 33.85, 33.55, 32.63, 32.40, 30.42, 29.83, 27.67, 27.60, 26.00, 25.59; HRMS (ESI$^+$) *m/z*: Calcd for $C_{22}H_{28}Br_4N$; [M+H]$^+$ 668.1100, Found 668.1101.

1-(6-(4-(4,5-dibromo-1-(6-morpholinohexyl)-1H-pyrrol-2-yl)-1H-imidazo[4,5-c]pyridin-1-yl)hexyl)morpholine (**27**)

Yellow adhesive liquid (84 mg, yield 83%). ^{1}H NMR (500 MHz, Chloroform-*d*) δ 8.38 (d, *J* = 5.5 Hz, 1H), 7.93 (s, 1H), 7.54 (s, 1H), 7.20 (d, *J* = 5.5 Hz, 1H), 4.74–4.64 (m, 2H), 4.18 (t, *J* = 7.1 Hz, 2H), 3.71 (s, 8H), 2.43 (s, 8H), 2.34–2.25 (m, 4H), 1.94–1.86 (m, 2H), 1.73–1.66 (m, 2H), 1.52–1.46 (m, 2H), 1.45–1.40 (m, 2H), 1.37 (s, 4H), 1.25 (s, 4H); ^{13}C NMR (125 MHz, Chloroform-*d*) δ 143.73, 143.31, 140.89, 139.21, 137.51, 130.18, 117.34, 108.69, 103.37, 99.03, 66.83, 66.75, 58.95, 58.77, 53.69, 53.62, 48.23, 45.21, 30.57, 29.83, 26.93, 26.88, 26.66, 26.29, 26.22, 26.08; HRMS (ESI$^+$) *m/z*: Calcd for $C_{30}H_{45}Br_2N_6O_2$; [M+H]$^+$ 679.1965, Found 679.1966.

1-(6-(4-(1-(6-((amino(iminio)methyl)thio)hexyl)-4,5-dibromo-1H-pyrrol-2-yl)-1H-imidazo[4,5-c]pyridin-1-yl)hexyl)isothiouronium hydrobromide (**28**)

Brown adhesive liquid (55 mg, yield 45%). ^{1}H NMR (400 MHz, Chloroform-*d*) δ 8.43 (d, *J* = 5.6 Hz, 1H), 8.40 (s, 1H), 7.67 (d, *J* = 5.6 Hz, 1H), 7.10 (s, 1H), 4.59 (t, *J* = 7.0 Hz, 2H), 4.41 (t, *J* = 7.1 Hz, 2H), 3.17 (t, *J* = 7.3 Hz, 2H), 3.03 (t, *J* = 7.3 Hz, 2H), 1.97 (dt, *J* = 14.5, 7.1 Hz, 2H), 1.74 (dt, *J* = 14.8, 7.4 Hz, 2H), 1.52 (m, 10H), 1.17 (dd, *J* = 14.6, 7.6 Hz, 2H); ^{13}C NMR (100 MHz, Chloroform-*d*) δ 171.61, 145.84, 142.53, 140.52, 139.97, 137.42, 130.21, 116.06, 107.64, 105.38, 98.53, 47.36, 44.99, 30.46, 30.33, 29.92, 29.38, 28.15, 27.86, 27.52, 27.29, 25.72, 25.07; HRMS (ESI$^+$) *m/z*: Calcd for $C_{24}H_{35}Br_2N_8S_2$; [M+H]$^+$ 657.0787, Found 657.0780.

4. **Figure S1**: Screening of compounds by a STAT3-based luciferase drug screening system.

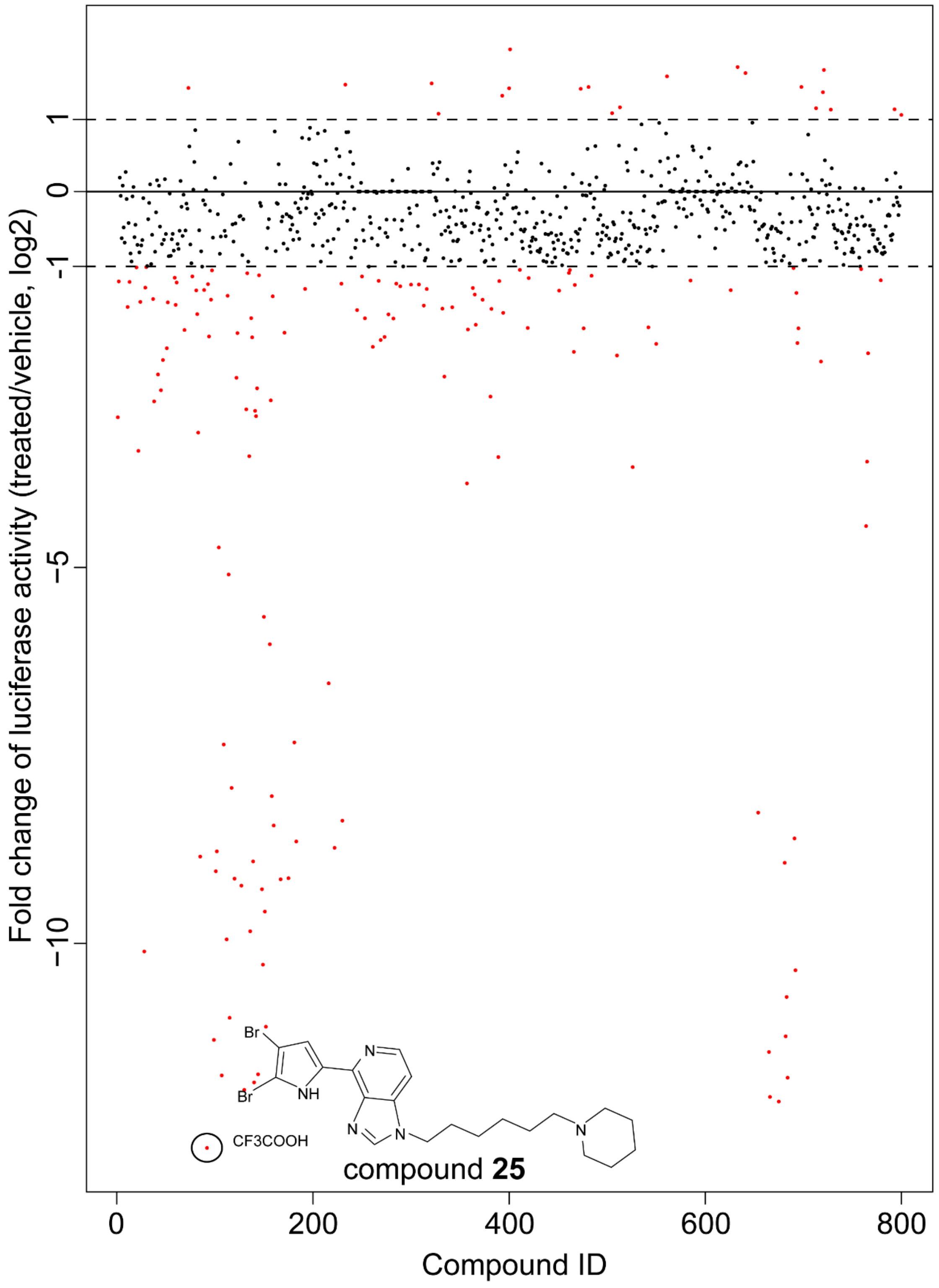

5. **Figure S2:** The result of cellular thermal shift assay (CETSA)

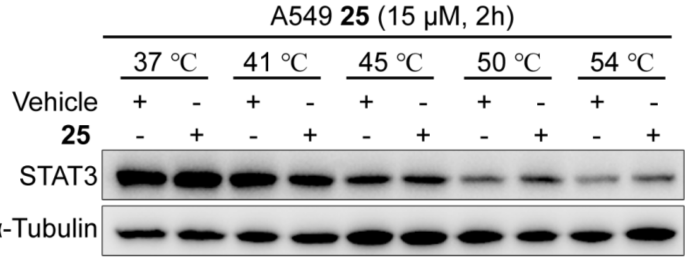

6. **Figure S3:** Quantification of Western blot

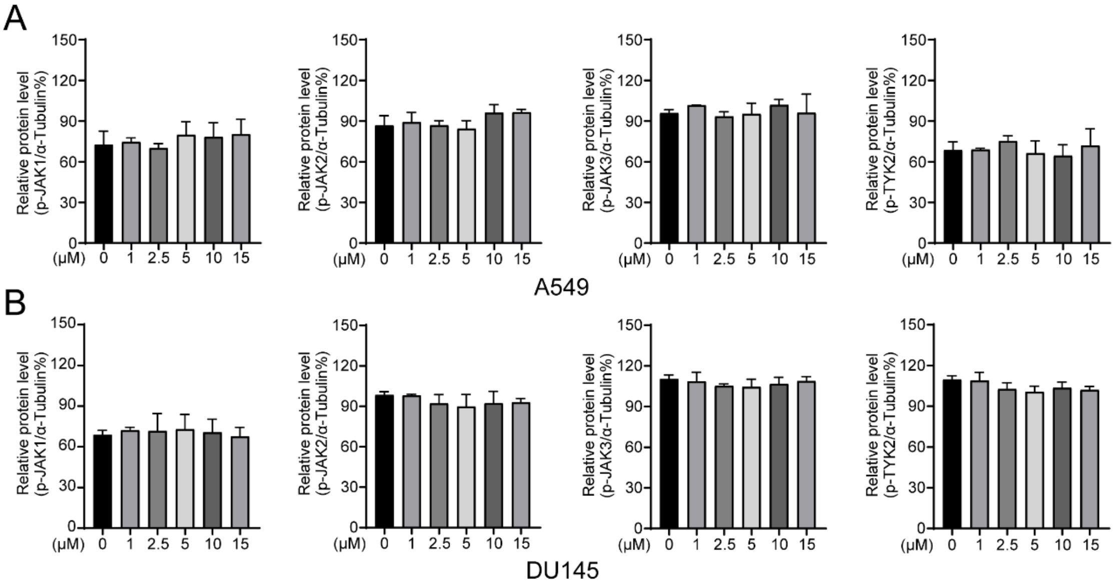

(A) Quantification for p-JAK/α-Tubulin in A549 cells in Figure 4A. Values are presented as mean ± SD. n = 3. (B) Quantification for p-JAK/α-Tubulin in DU145 cells in Figure 4B. Values are presented as mean ± SD. n = 3.

7. **Figure S4:** Quantification of Western blot

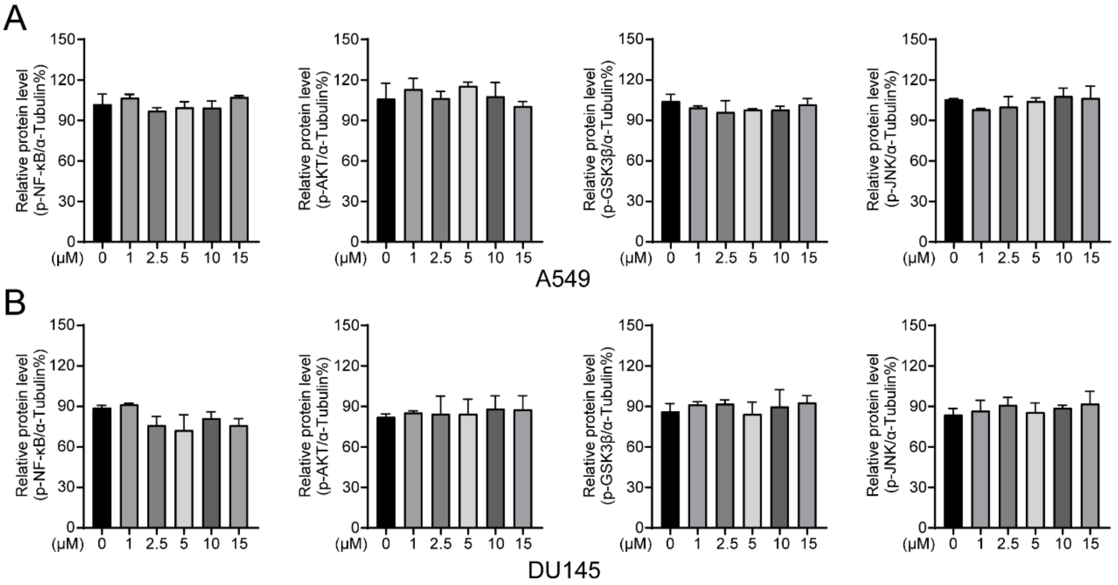

(A) Quantification for p-NF-κB/α-Tubulin, p-AKT/α-Tubulin, p-GSK3β/α-Tubulin, and p-JNK/α-Tubulin in A549 cells in Figure 4C. Values are presented as mean ± SD. n = 3. (B) Quantification

for p-NF-κB/α-Tubulin, p-AKT/α-Tubulin, p-GSK3β/α-Tubulin, and p-JNK/α-Tubulin in A549 cells in Figure 4D. Values are presented as mean ± SD. n = 3.

8. **Figure S5:** Quantification of Western blot

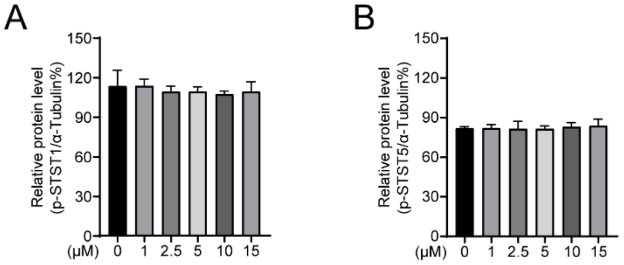

(A) Quantification for p-STAT1/α-Tubulin in A549 cells in Figure 4E. Values are presented as mean ± SD. n = 3. (B) Quantification for p-STAT5/α-Tubulin in A549 cells in Figure 4E. Values are presented as mean ± SD. n = 3.

9. **Figure S6:** Analysis diagram of liquid-purity of Compound **25**.

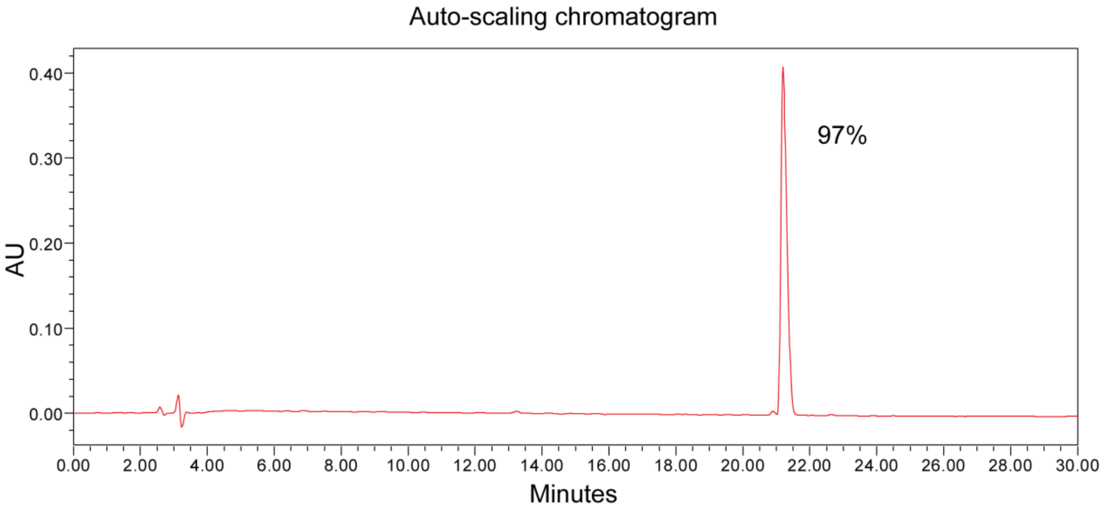

10. The data of ^{1}H and ^{13}C NMR of all the compounds.

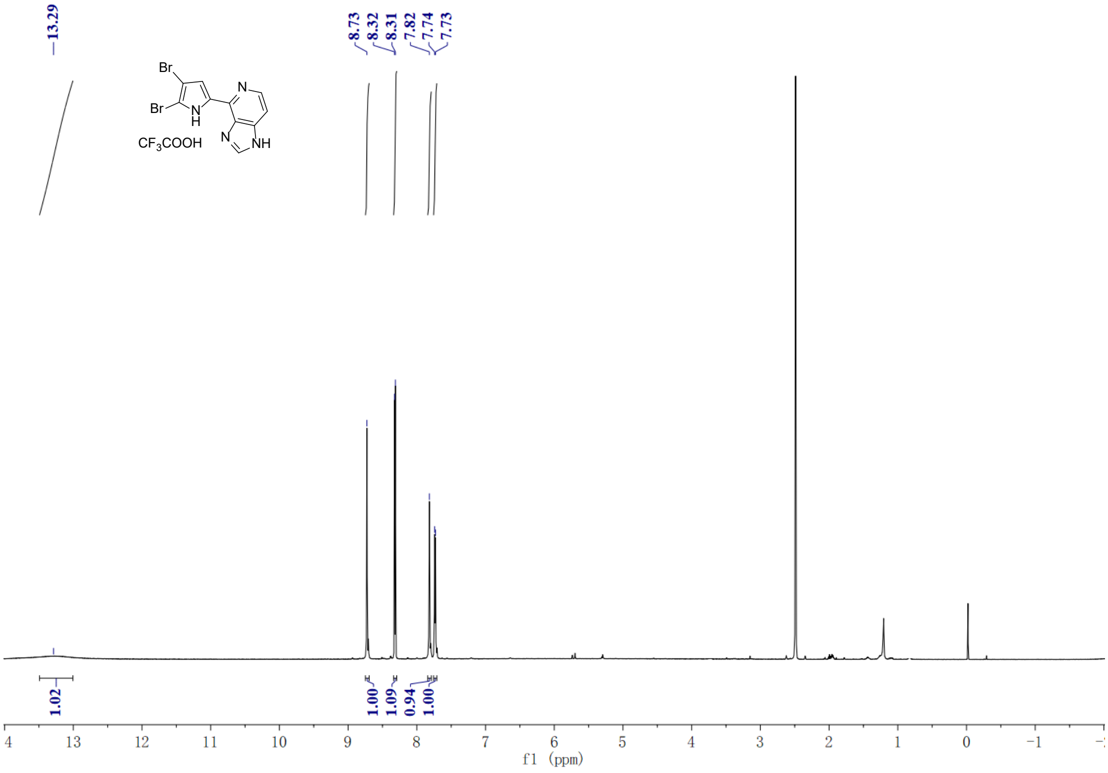

^{1}H NMR Spectrum of **11** in DMSO-d_6

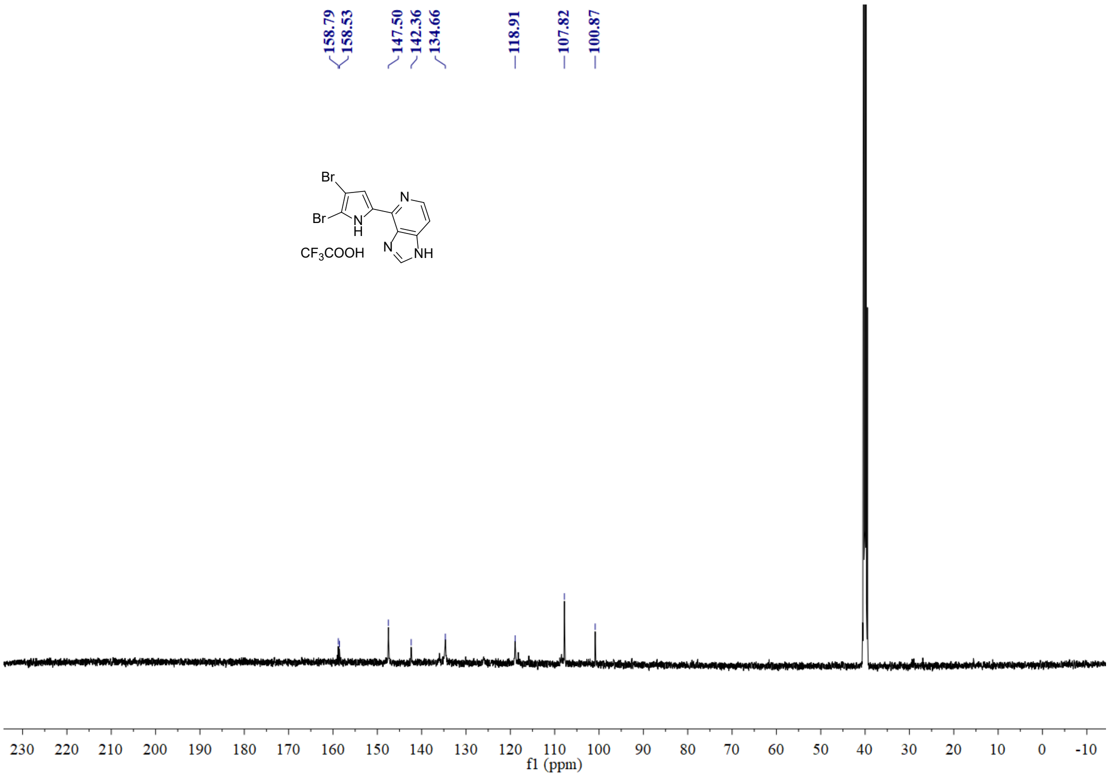

^{13}C NMR Spectrum of **11** in DMSO-d_6

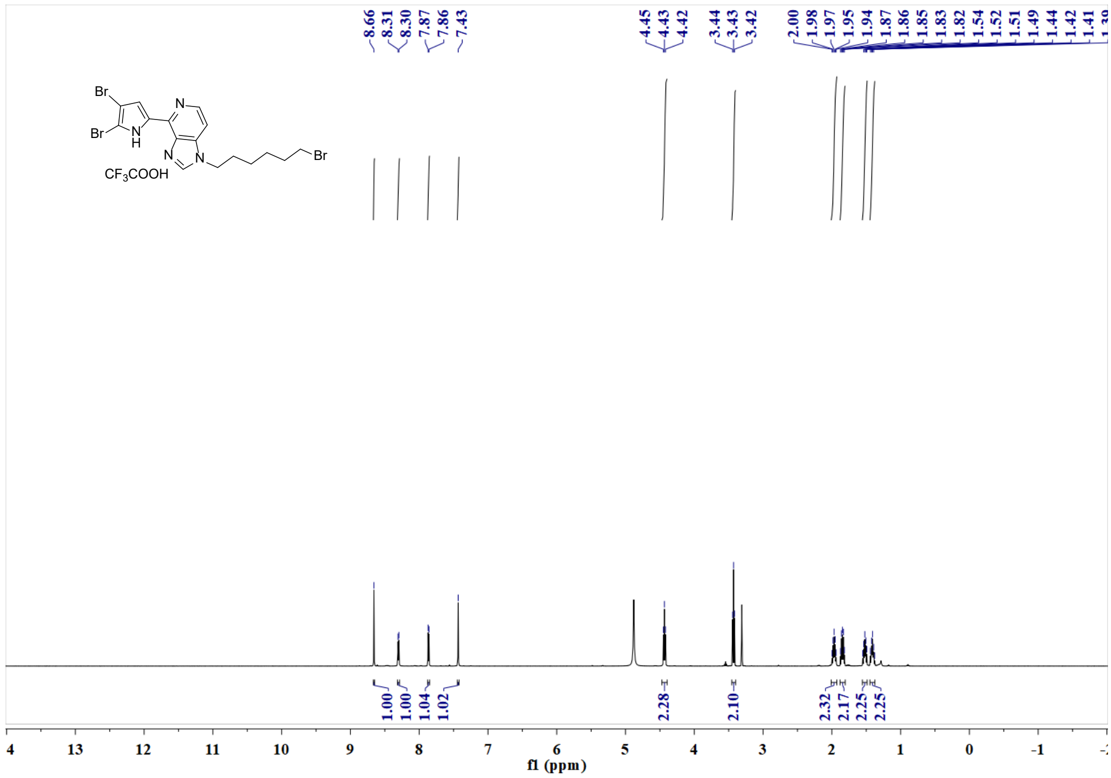

^{1}H NMR Spectrum of **12** in DMSO-d_6

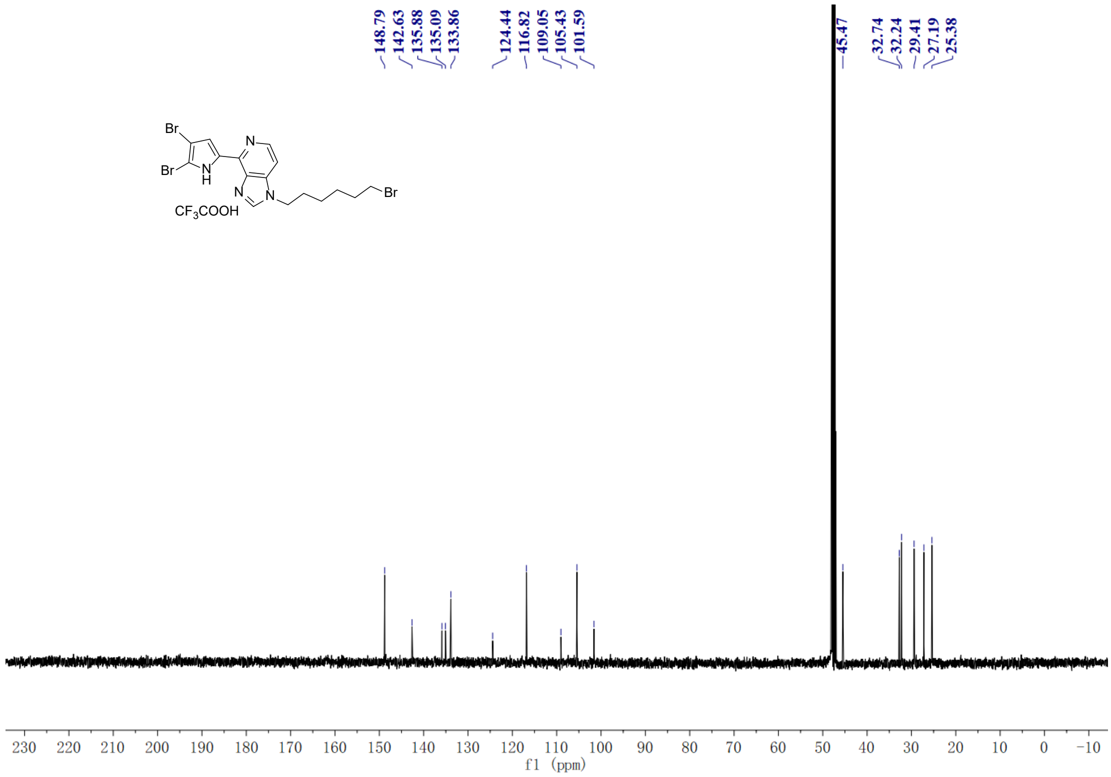

^{13}C NMR Spectrum of **12** in DMSO-d_6

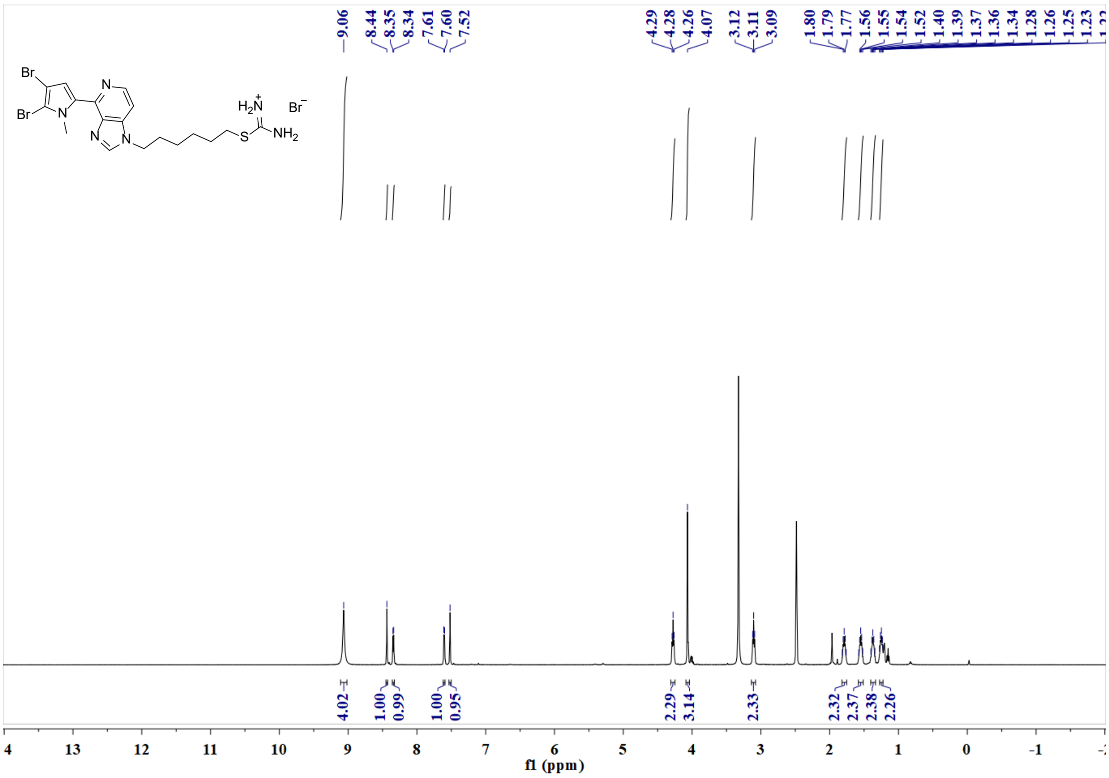

^{1}H NMR Spectrum of **14** in DMSO-d_6

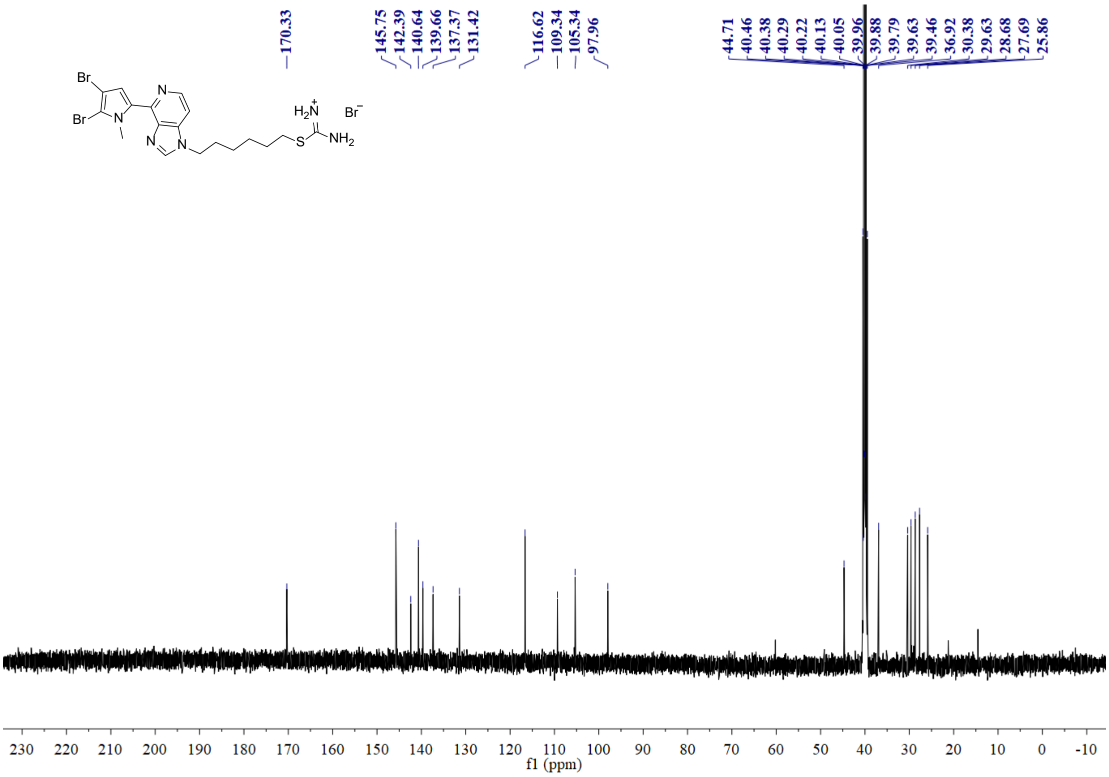

^{13}C NMR Spectrum of **14** in DMSO-d_6

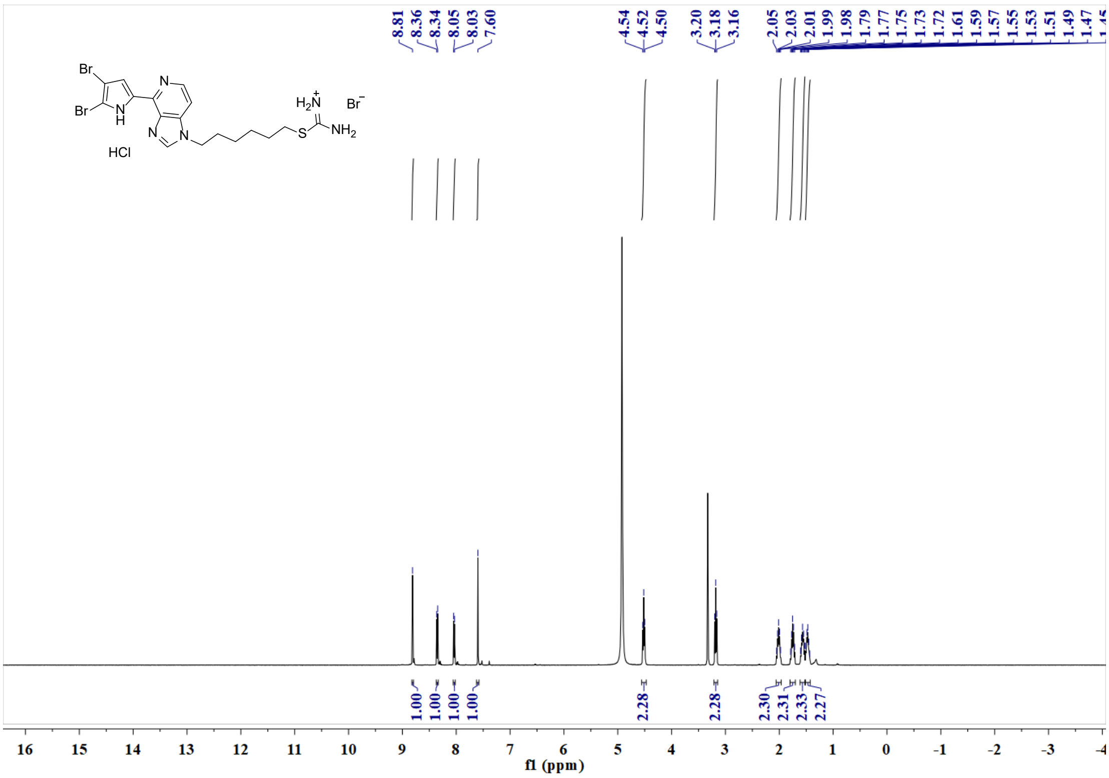

^{1}H NMR Spectrum of **15** in Methanol-d_4

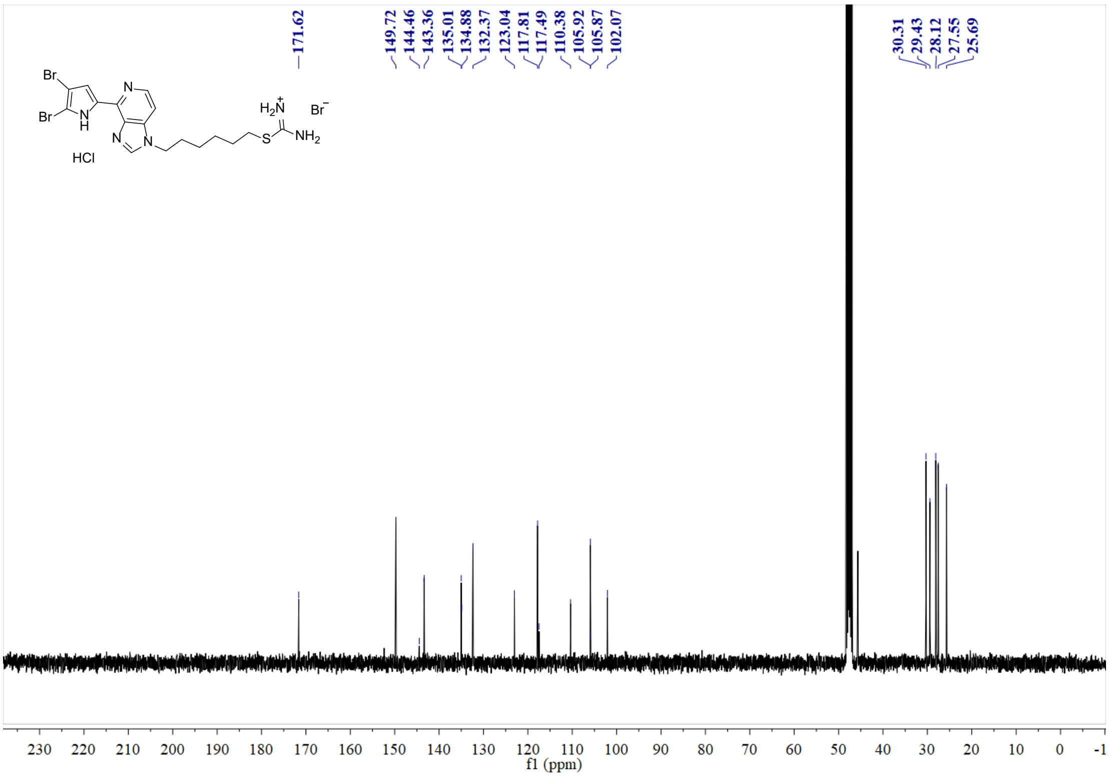

^{13}C NMR Spectrum of **15** in Methanol-d_4

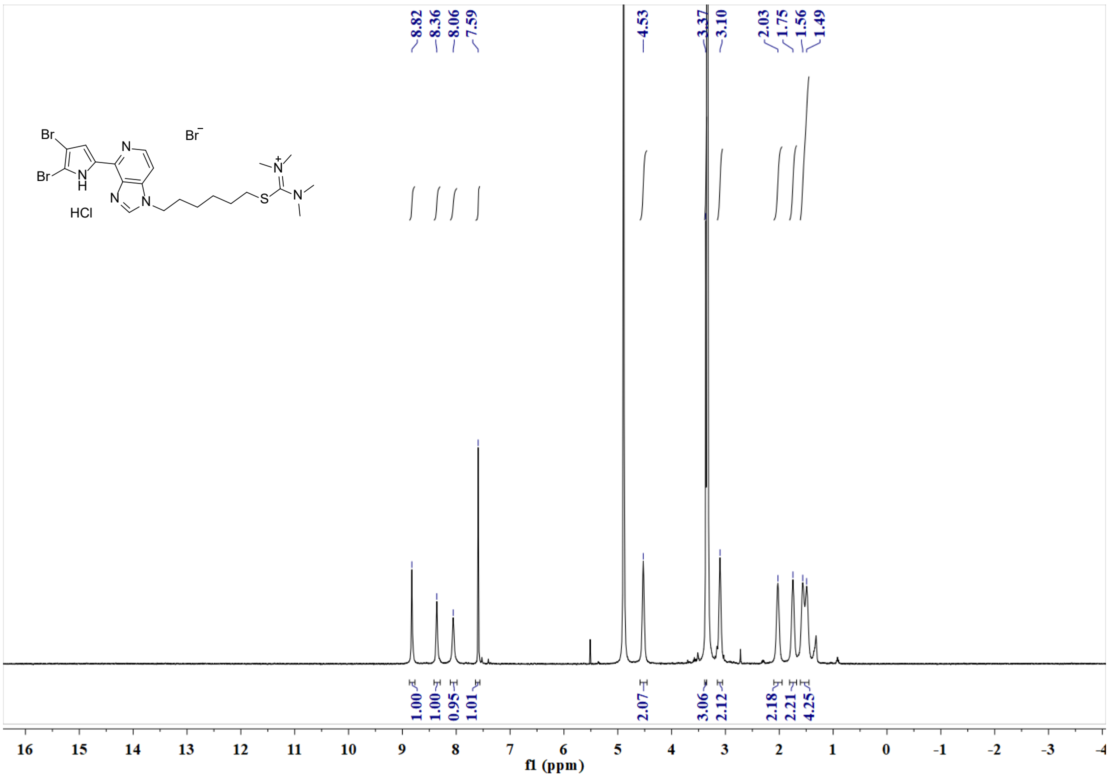

^{1}H NMR Spectrum of **17** in Methanol-d_4

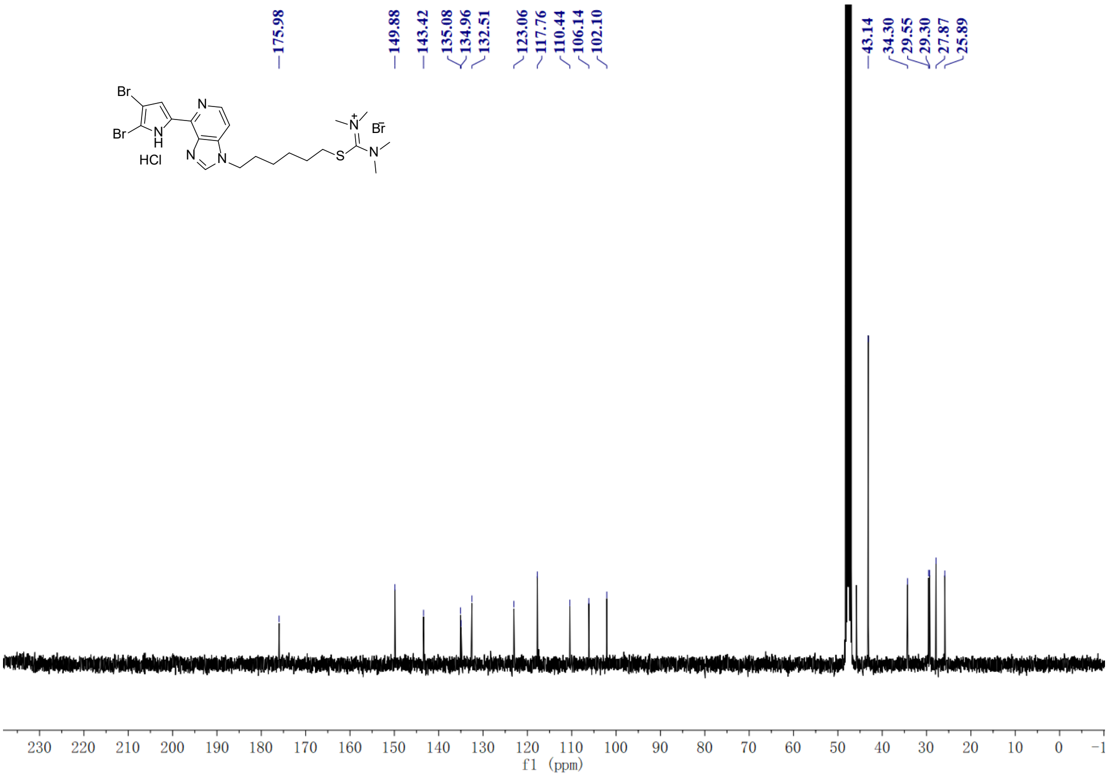

^{13}C NMR Spectrum of **17** in Methanol-d_4

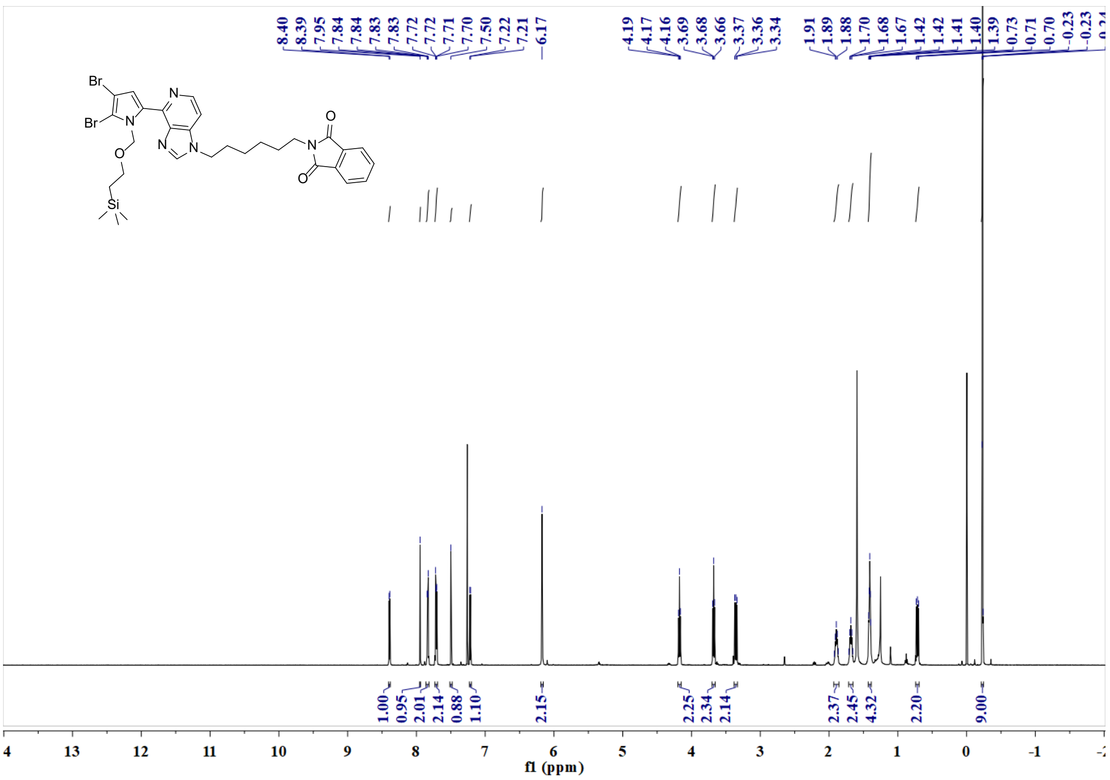

^{1}H NMR Spectrum of **20** in Chloroform-*d*

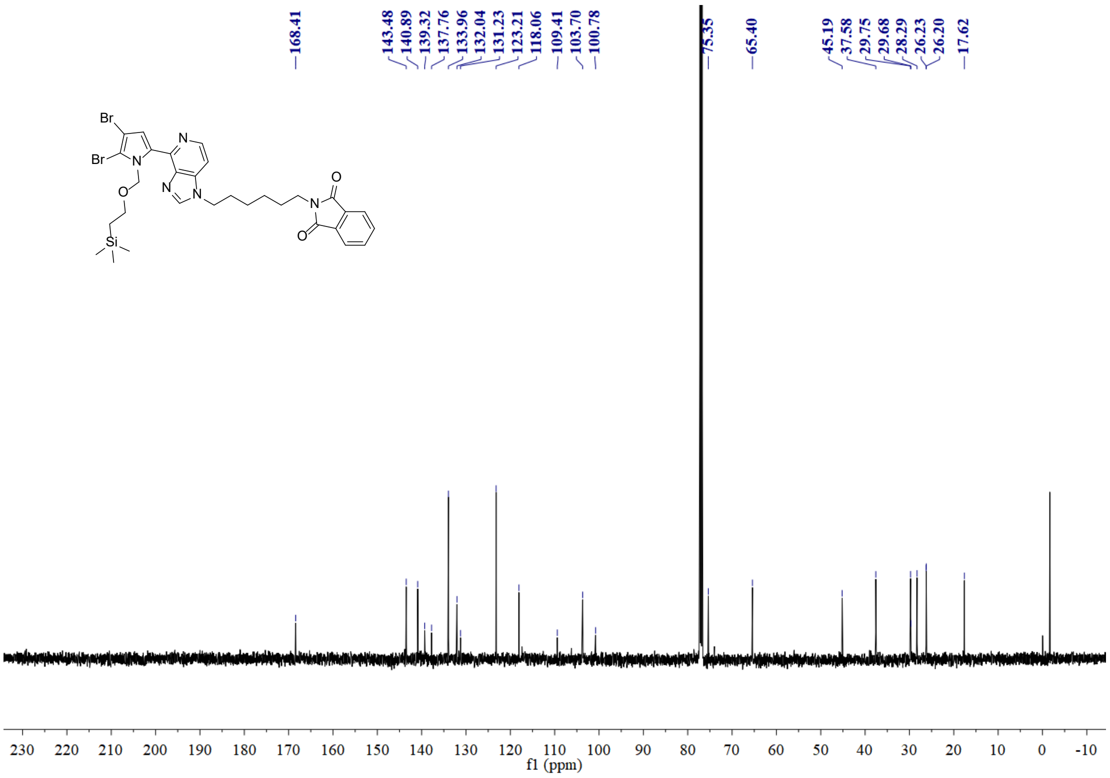

^{13}C NMR Spectrum of **20** in Chloroform-*d*

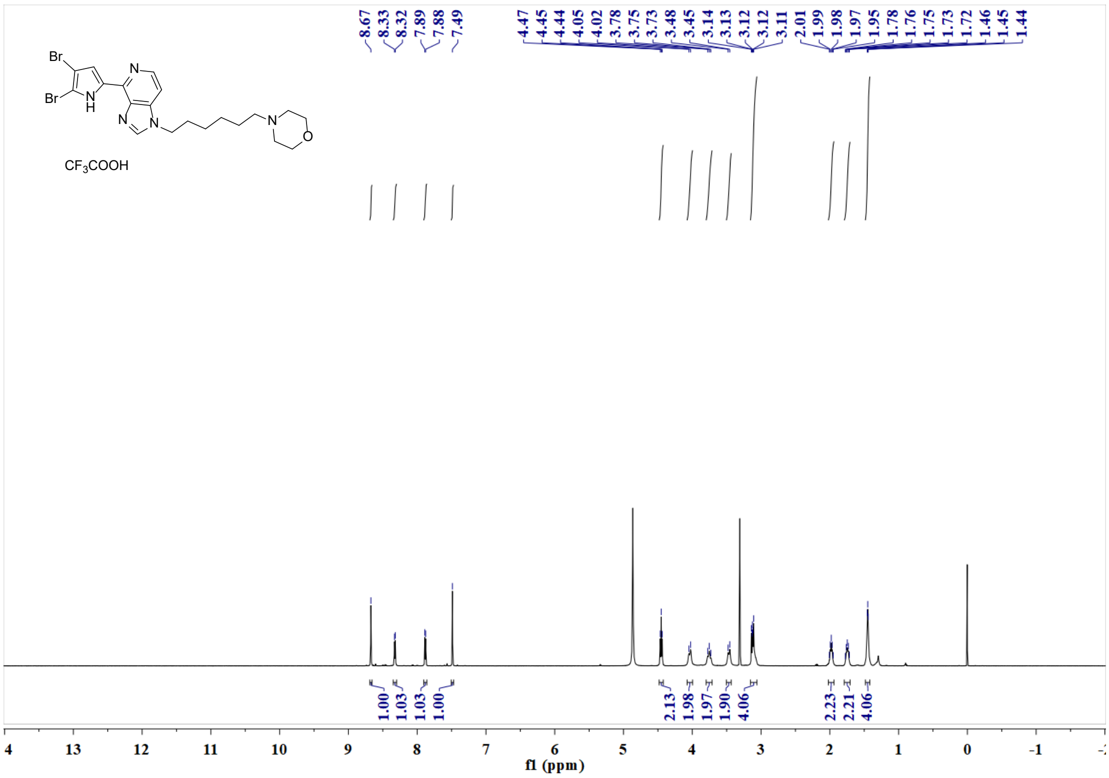

^{1}H NMR Spectrum of **22** in Methanol-d_4

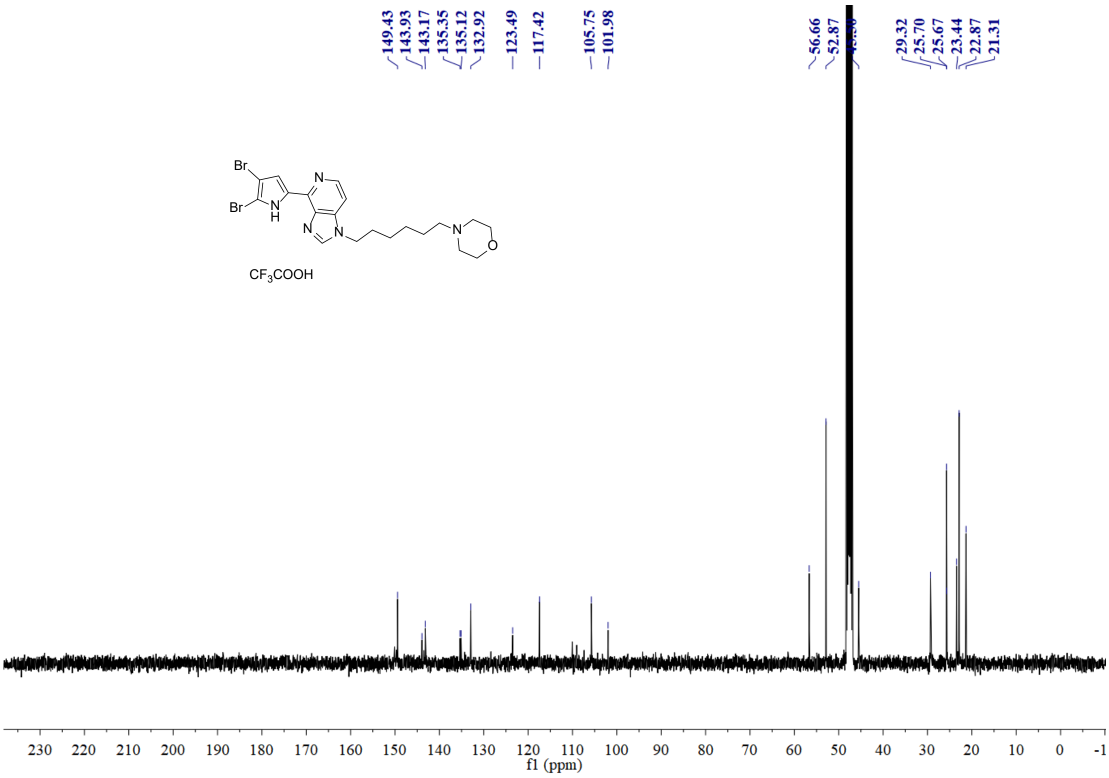

^{13}C NMR Spectrum of **22** in Methanol-d_4

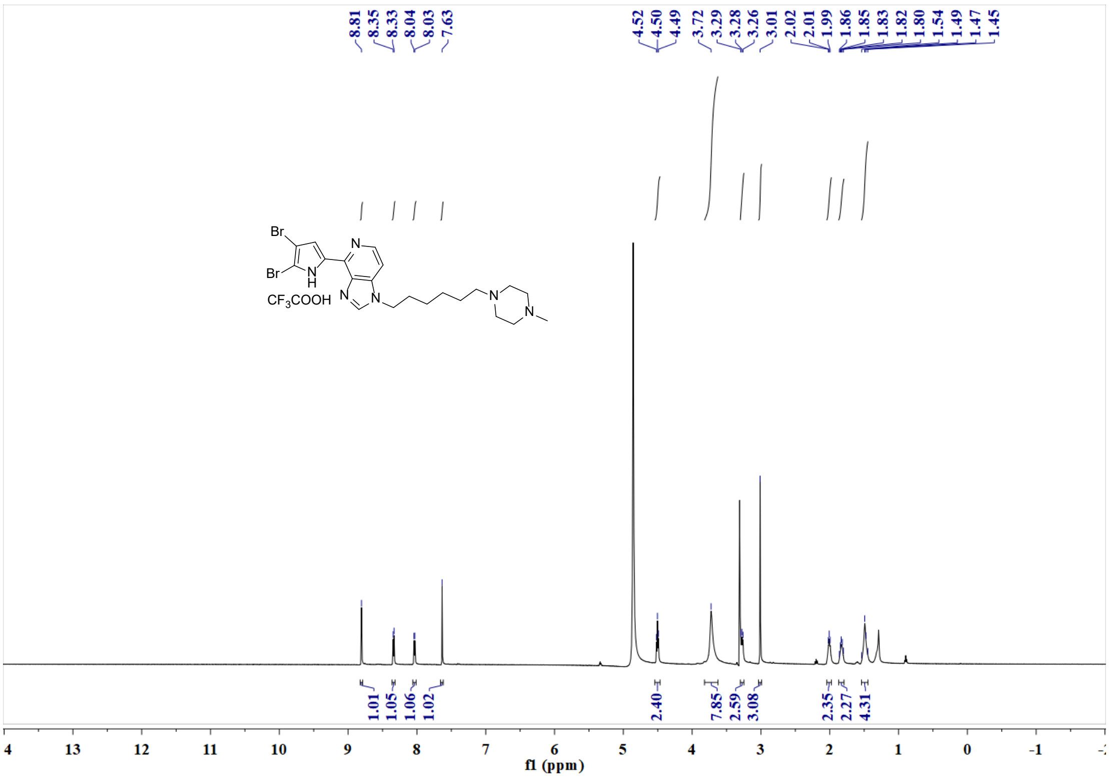

^{1}H NMR Spectrum of **23** in Methanol-d_4

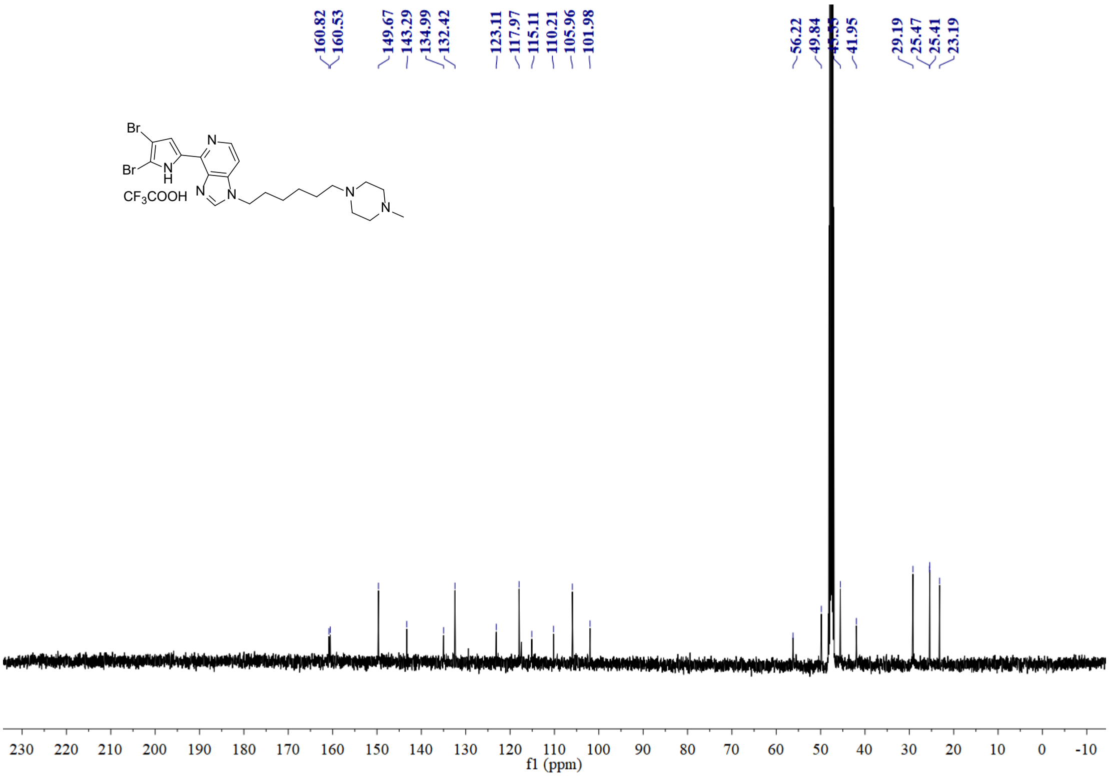

^{13}C NMR Spectrum of **23** in Methanol-d_4

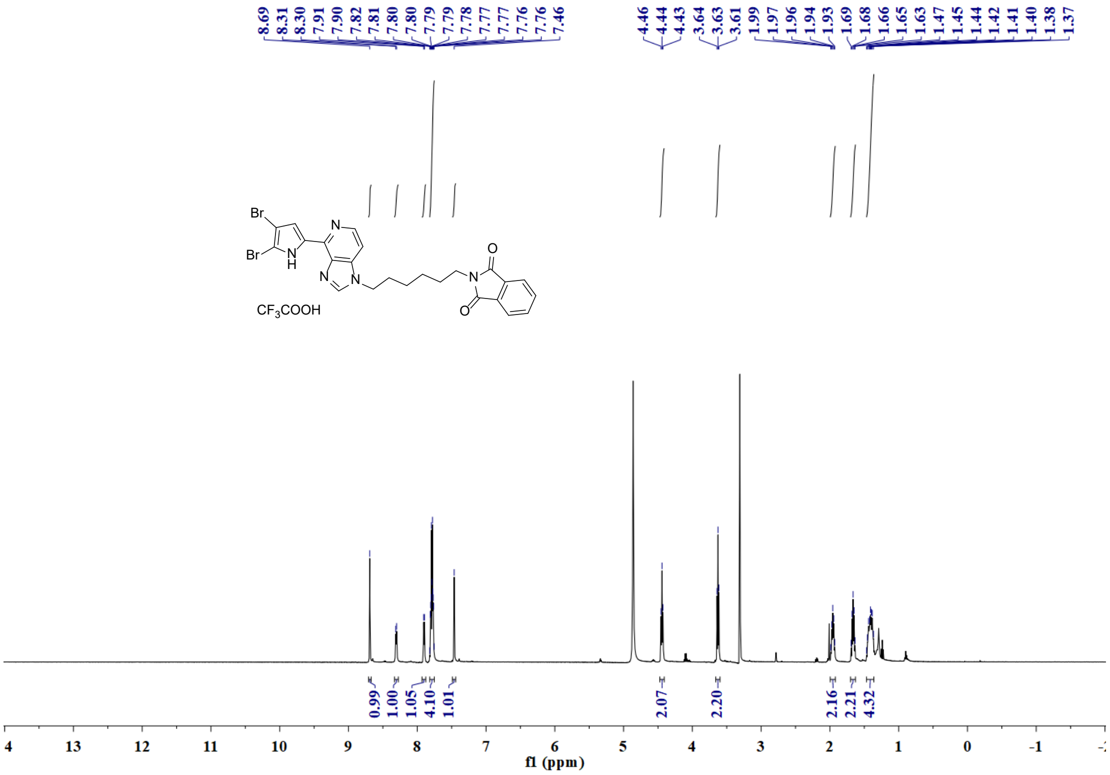

^{1}H NMR Spectrum of **24** in Methanol-*d*$_4$

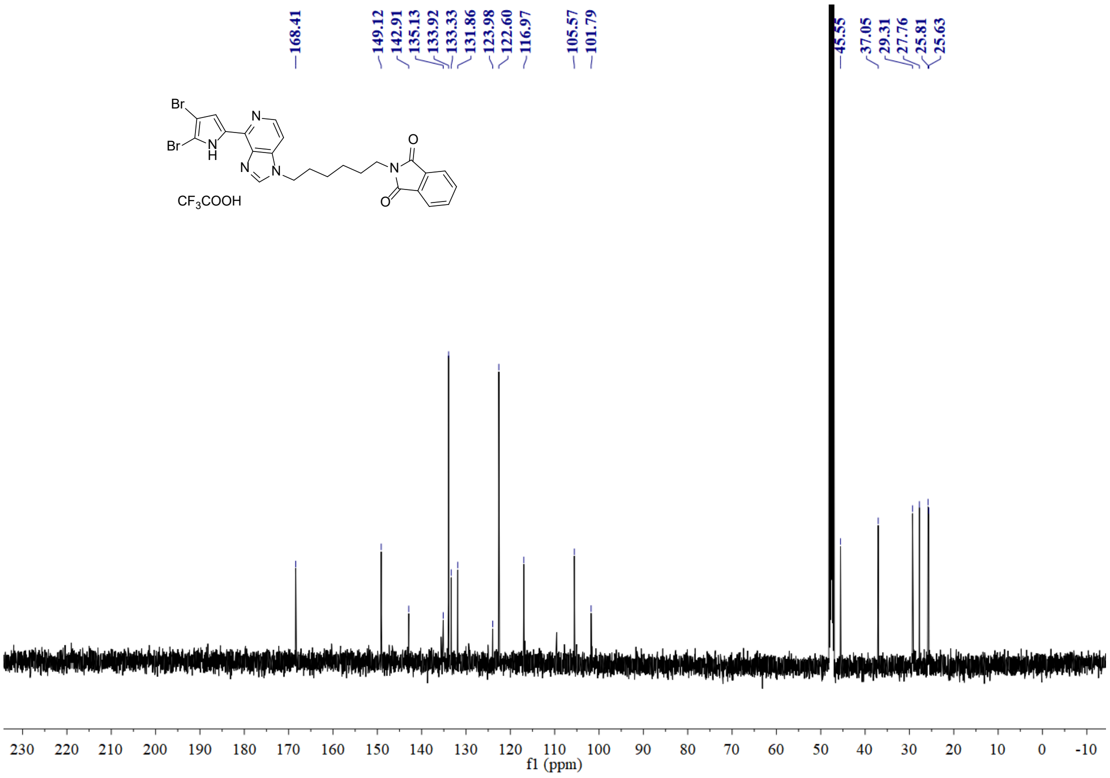

^{13}C NMR Spectrum of **24** in Methanol-*d*$_4$

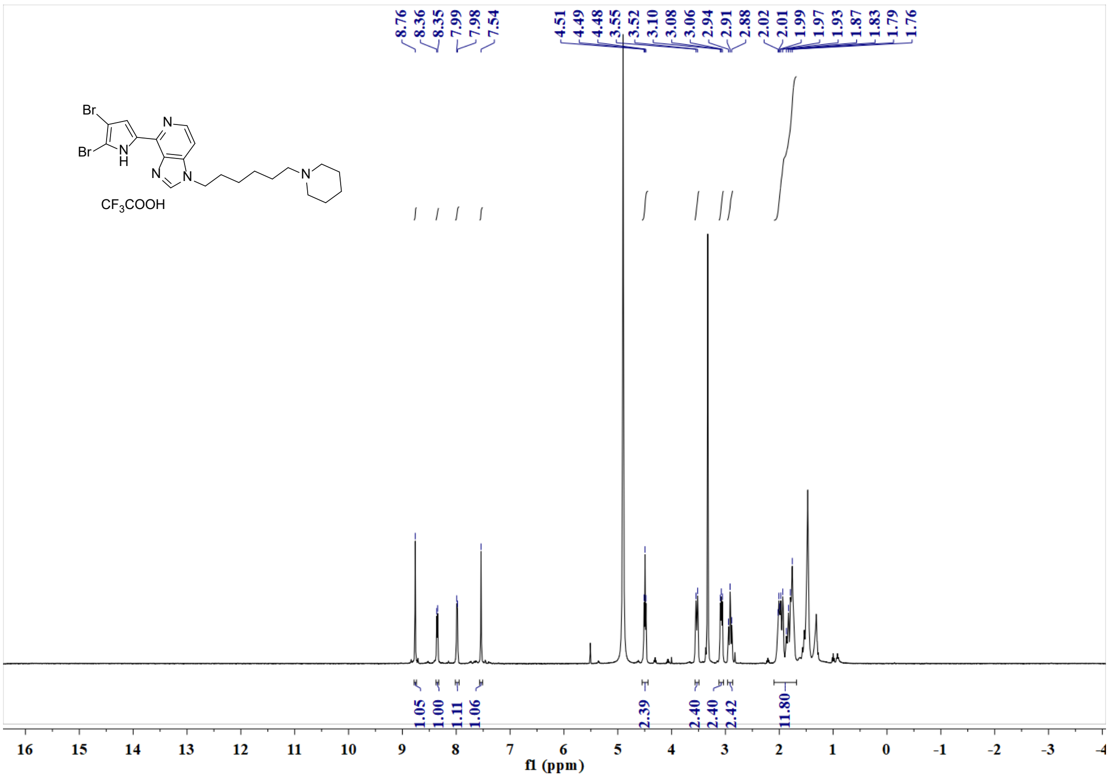

^{1}H NMR Spectrum of **25** in Methanol-d_4

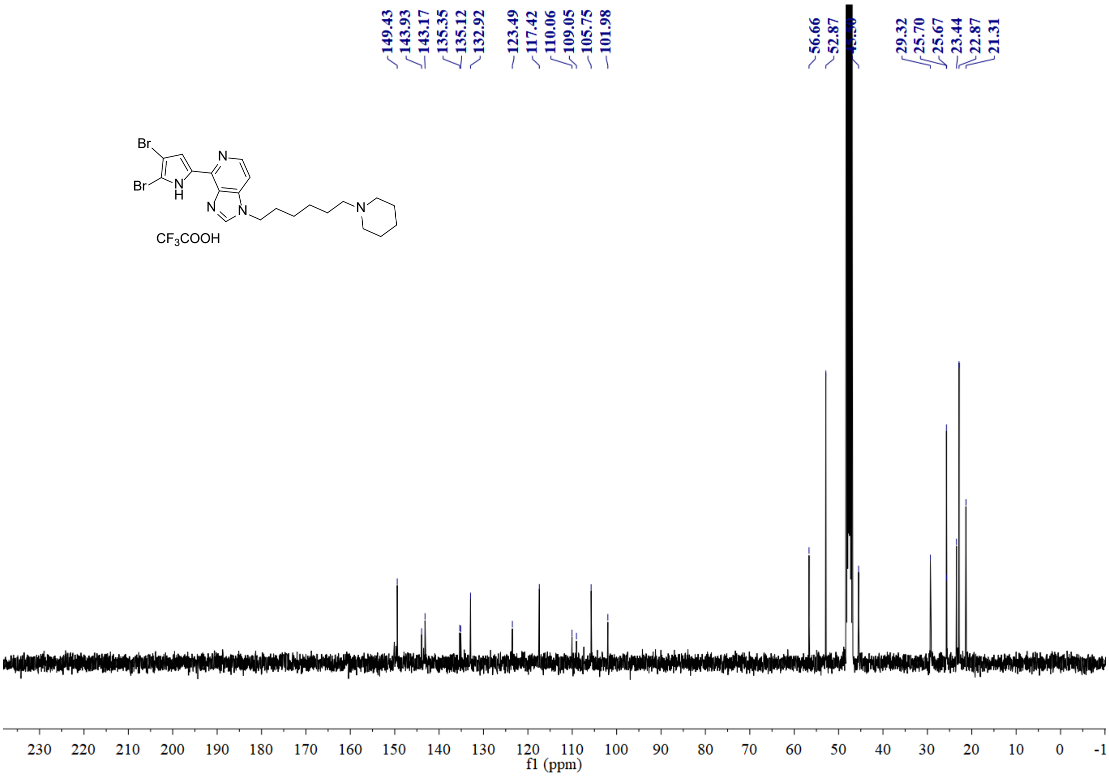

^{13}C NMR Spectrum of **25** in Methanol-d_4

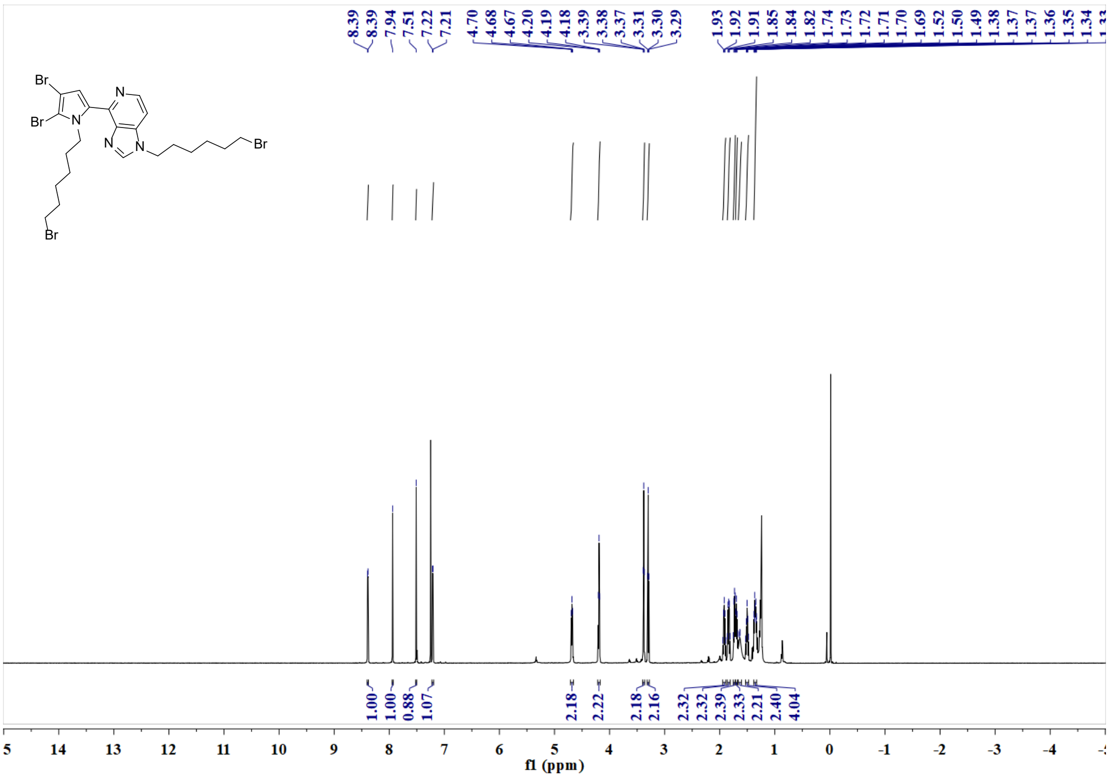

^{1}H NMR Spectrum of **26** in Chloroform-*d*

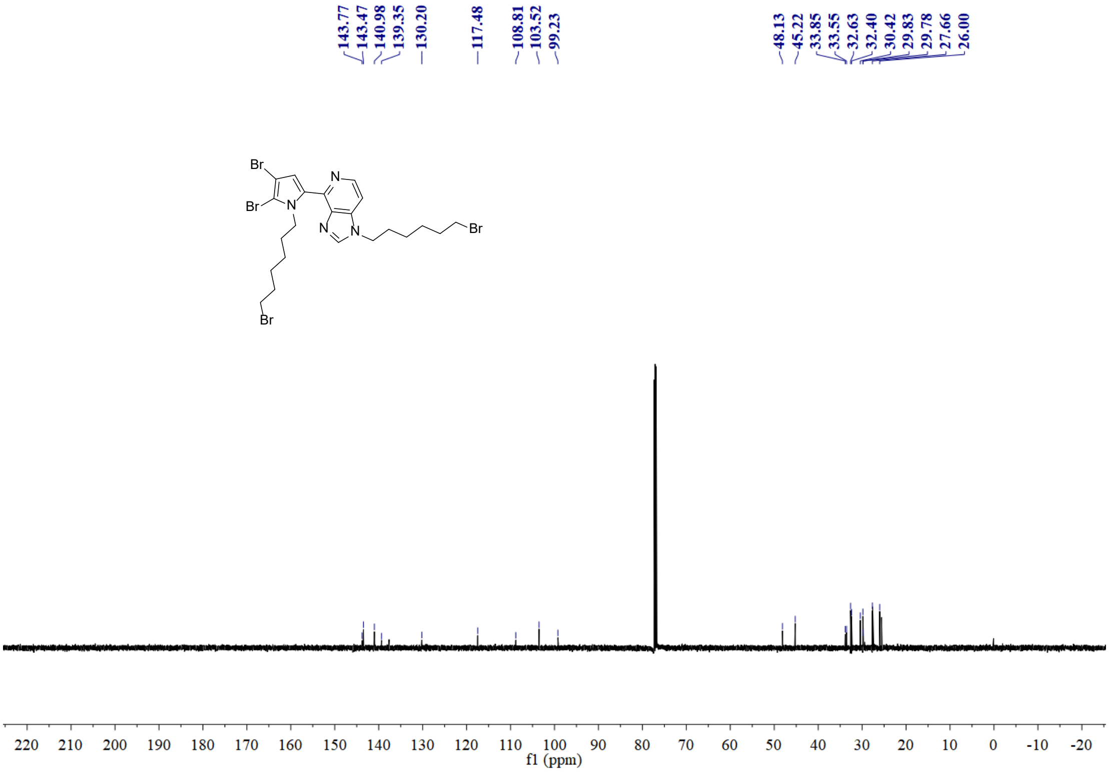

^{13}C NMR Spectrum of **26** in Chloroform-*d*

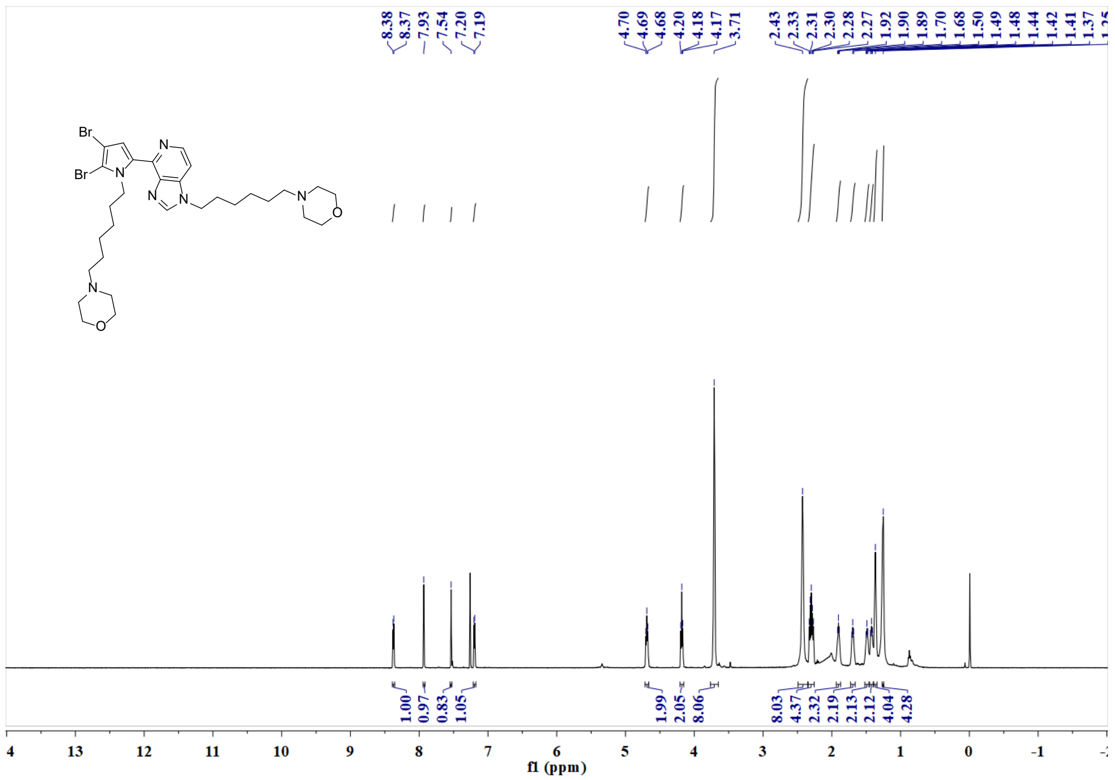

^{1}H NMR Spectrum of **27** in Chloroform-*d*

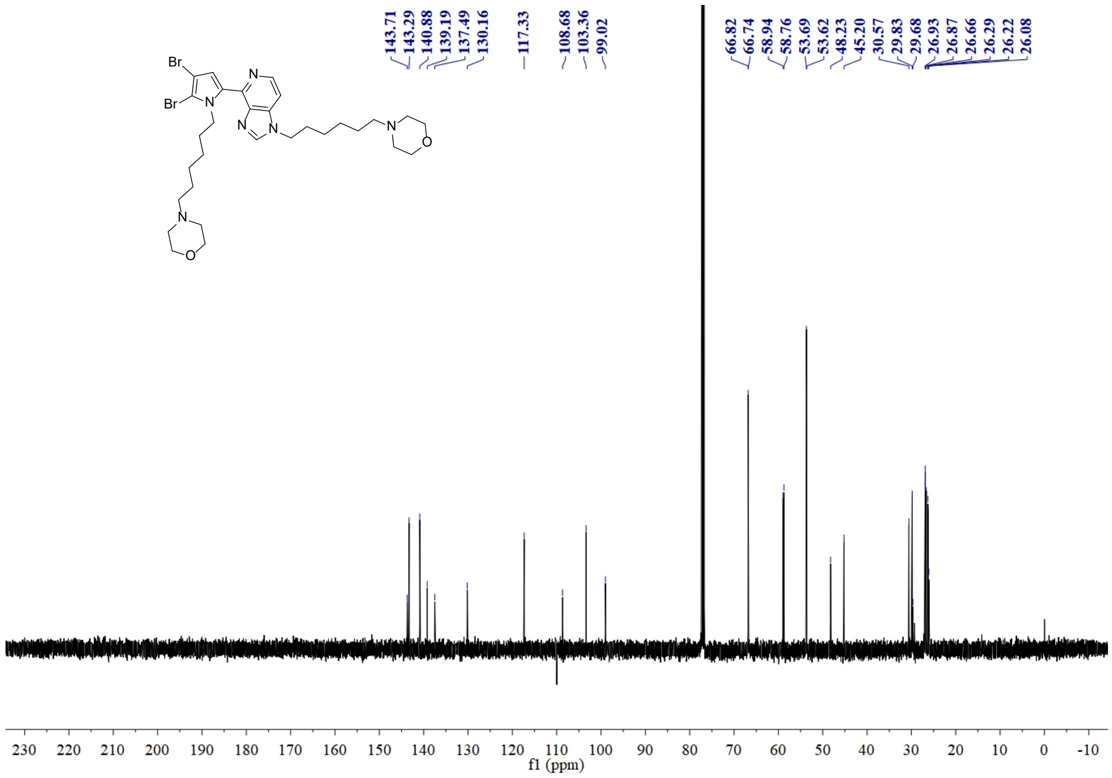

^{13}C NMR Spectrum of **27** in Chloroform-*d*

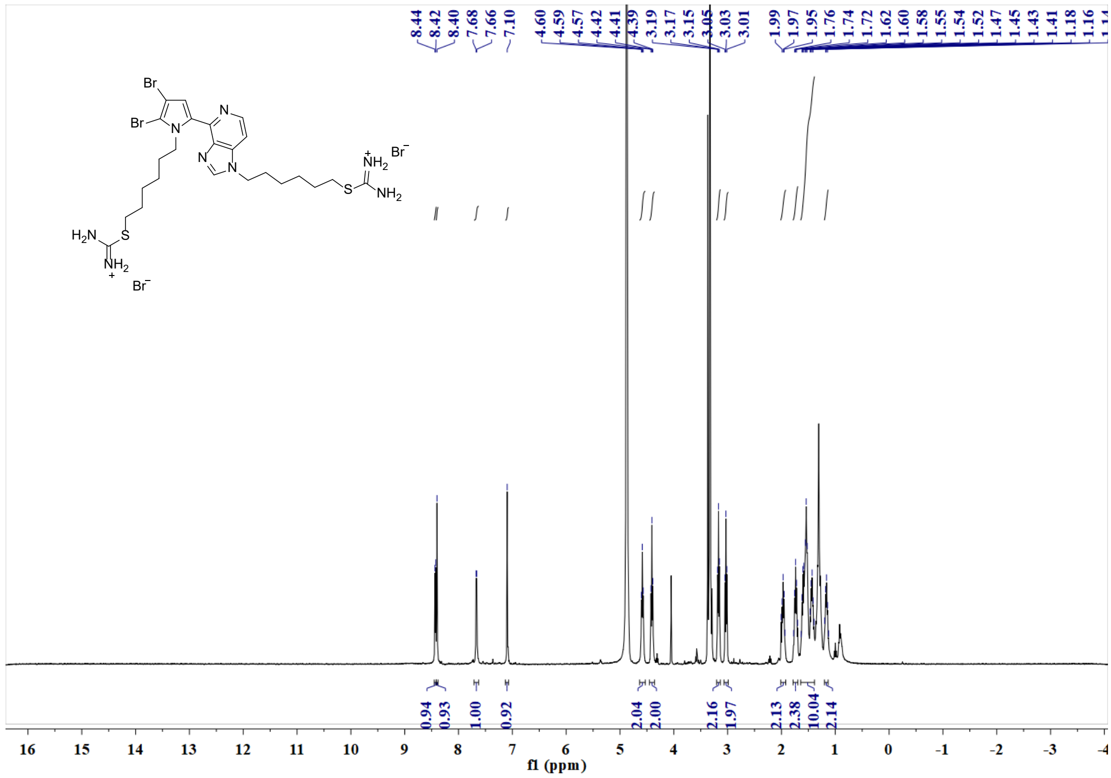

^{1}H NMR Spectrum of **28** in Methanol-d_4

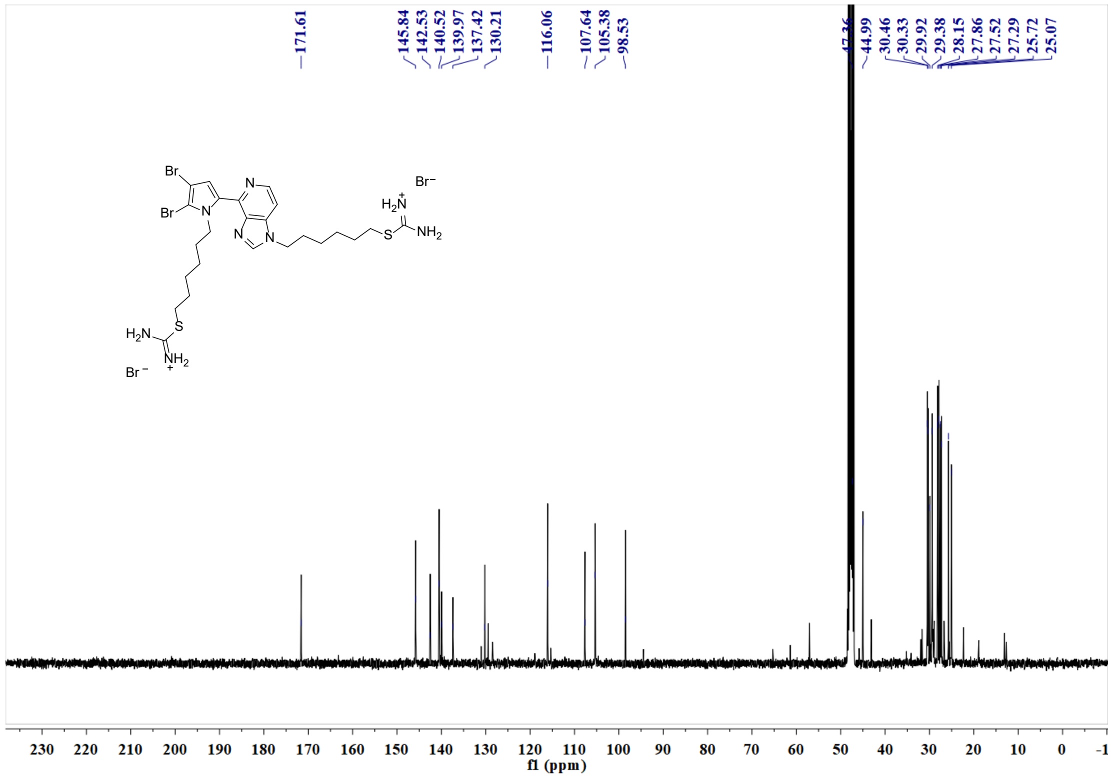

^{13}C NMR Spectrum of **28** in Methanol-d_4

[34]. Zhang, Y.; Banwell, M.G.; Carr, P.D.; Willis, A.C. Modular Total Syntheses of the Alkaloids Discoipyrroles A and B, Potent Inhibitors of the DDR2 Signaling Pathway. Org. Lett. 2016, 18, 704 – 707.
